# Association between glucagon-like peptide-1 receptor agonist therapy and respiratory illness in patients with type 2 diabetes: a retrospective observational cohort study

**DOI:** 10.1038/s41598-025-19657-5

**Published:** 2025-10-13

**Authors:** Li-Ti Ho, Yu-Wei Fang, Pei-Sung Hsu, Jing-Tong Wang, Ming-Hsien Tsai

**Affiliations:** 1 Department of General Medicine, Shin Kong Wu Ho-Su Memorial Hospital, No. 95, Wen-Chang Rd, Shih-Lin Dist., Taipei, 11101 Taiwan; 2 Department of Medicine, Fu-Jen Catholic University School of Medicine, No. 510, Zhongzheng Rd., Xinzhuang Dist., New Taipei City, 242062 Taiwan; 3 Division of Nephrology, Department of Internal Medicine, Shin Kong Wu Ho-Su Memorial Hospital, No. 95, Wen-Chang Rd, Shih-Lin Dist., Taipei, 11101 Taiwan; 4 Division of Pulmonology, Department of Internal Medicine, Shin Kong Wu Ho-Su Memorial Hospital, No. 95, Wen-Chang Rd, Shih-Lin Dist., Taipei, 11101 Taiwan; 5 Department of Healthcare Management, National Taipei University of Nursing and Health Sciences, No. 89, Neijiang St., Wanhua Dist., Taipei City, 108306 Taiwan; 6 Data Analysis Group, Department of Digital Medicine, Shin-Kong Wu Ho-Su Memorial Hospital, No. 95, Wen-Chang Rd, Shih-Lin Dist., Taipei, 11101 Taiwan

**Keywords:** Type 2 diabetes mellitus, Glucagon-like peptide-1 receptor agonist, Dipeptidyl peptidase-4 inhibitor, Lung cancer, Real-world evidence, Diseases, Endocrinology, Medical research

## Abstract

Studies have shown that glucagon-like peptide-1 receptor agonists (GLP-1 RAs) provide lung benefits beyond glycemic control, including reduced risks of lung malignancy, pulmonary infection and chronic obstructive pulmonary disease exacerbations. A retrospective cohort study with a new-user and active-comparator design was conducted using the TriNetX US Network. Adults (≥ 18 years) with Type 2 diabetes mellitus (T2DM) (*n* = 3,389,059) from January 1, 2005, to December 31, 2020, were identified. Two cohorts of new GLP-1 RA (*n* = 201,153) and new dipeptidyl peptidase-4 inhibitor (DPP4i) (*n* = 323,114) users were created. Propensity score matching was used to create comparable cohorts. The primary outcome was lung cancer, while other pulmonary conditions were secondary outcomes over a 10-year follow-up. Through 1:1 propensity score matching, two balanced cohorts of GLP-1 RA and DPP4i (*n* = 158,224) users were created. GLP-1 RA users had a significantly lower risk of lung cancer than did DPP4i users (hazard ratios [HR] 0.86; 95% confidence interval [CI] 0.80–0.94). Reduced risk of influenza and pneumonia (HR 0.94; 95% CI 0.92–0.96) and pulmonary fibrosis (HR 0.92; 95% CI 0.87–0.98) were also noted in GLP-1 RA users. Our findings indicate that GLP-1 RAs may lower the risks of lung cancer, pulmonary fibrosis, and respiratory infections, meriting further prospective study.

**Supplementary Information:**

The online version contains supplementary material available at 10.1038/s41598-025-19657-5.

## Background

Type 2 diabetes mellitus (T2DM) is a major global health issue and contributing to increasing rates of morbidity and mortality^1^. Traditionally, the management of T2DM has focused on glycemic control to prevent vascular complications^2^. However, the recognition that diabetes impacts a wide range of organ systems has broadened the focus to include the extra-glycemic effects of antidiabetic therapies^3^. Among these, glucagon-like peptide-1 receptor agonists (GLP-1 RAs) have become recognized as one of the promising categories of medications^4^.

GLP-1 RAs were originally developed for their ability to enhance glucose-dependent insulin secretion and support weight loss^4^ but accumulating evidence has revealed their beneficial effects on cardiovascular^5,6^, renal^7,8^ and hepatic outcomes^9,10^. These wide-ranging effects are believed to result from the presence of GLP-1 receptors in multiple organ systems, including the heart, kidneys, and liver^11^. Moreover, research has identified higher GLP-1 receptors in pulmonary tissues compared with other organs, suggesting that these therapies may also play a role in respiratory health^12^.

Interest in the impact of GLP-1 RAs on lung diseases has increased, particularly in light of studies reporting reduced risks of lung cancer^13–15^ asthma exacerbations^16^ chronic obstructive pulmonary disease (COPD) progression^17,18^ and overall lung illness^19^ among patients using these medications. The underlying mechanisms are not yet fully understood, but may involve anti-inflammatory actions, modulation of immune responses, and reduction of tissue injury and fibrosis^20,21^. Despite these promising findings, existing research on the association between GLP-1 RA use and respiratory outcomes remains limited in scope. Most studies to date have had small samples, brief follow-ups, or lacked direct comparisons, limiting definitive conclusions.

Given this unmet need, our study aims to provide a more comprehensive assessment of the potential respiratory benefits of GLP-1 RAs over a 10-year follow-up. Using a large, retrospective cohort from the TriNetX US electronic health record database, we compared the incidence of lung cancer, respiratory infections, and pulmonary fibrosis in adults with T2DM initiating GLP-1 RAs versus those using other commonly prescribed antihyperglycemic agents.

## Methods

### Data source

TriNetX (Cambridge, MA, USA) is a global health research consortium that includes health-care organizations, researchers, and biopharmaceutical enterprises. This platform facilitates clinical research and supports efforts to improve clinical outcomes by providing access to deidentified patient data from diverse health-care institutions, enabling comprehensive real-world data analysis^22^. TriNetX is designed to generate real-world evidence through the analysis of electronic health records, claims data, and other data sets. The TriNetX network includes more than 220 health-care organizations across over 30 countries, predominantly large academic medical centers but also community hospitals, most with both inpatient and outpatient services. For this study, only structured electronic health record (EHR) data (demographics, diagnoses, procedures, prescriptions, laboratories, and vital signs) were available; unstructured records and linkages to external claims or cancer registries were not accessible. Routine TriNetX datasets do not include claims data, and while some institutions may internally link EHR data with claims before contributing, this is neither standardized nor common across the network. Likewise, direct linkage to external cancer registries is uncommon, although some sites may connect EHR data to their own hospital-based registries; this practice is also not consistent network-wide. The platform allows investigators to identify and analyze patient populations for clinical trials, streamlining the recruitment process. Furthermore, the platform provides advanced data visualization and analytical tools, enabling users to explore trends, treatment modalities, and outcomes. All data within TriNetX are de-identified, ensuring that patient identifiers are not accessible. The platform aggregates data counts and summary statistics from multiple institutions without including individual-level data. Given these safeguards, the Western Institutional Review Board has waived the requirement for informed consent for studies using TriNetX data.

### Ethics approval and consent to participate

This study adhered to the International Conference on Harmonization Good Clinical Practice guidelines, the Declaration of Helsinki, and applicable laws for noninterventional and observational studies. The study protocol was approved by the Ethics Review Board of Shin Kong Wu Ho-Su Memorial Hospital, Taiwan (protocol number: 20250104R). The requirement for informed consent was waived because all personal information was deidentified.

### Study design and cohort

**Fig. 1:**
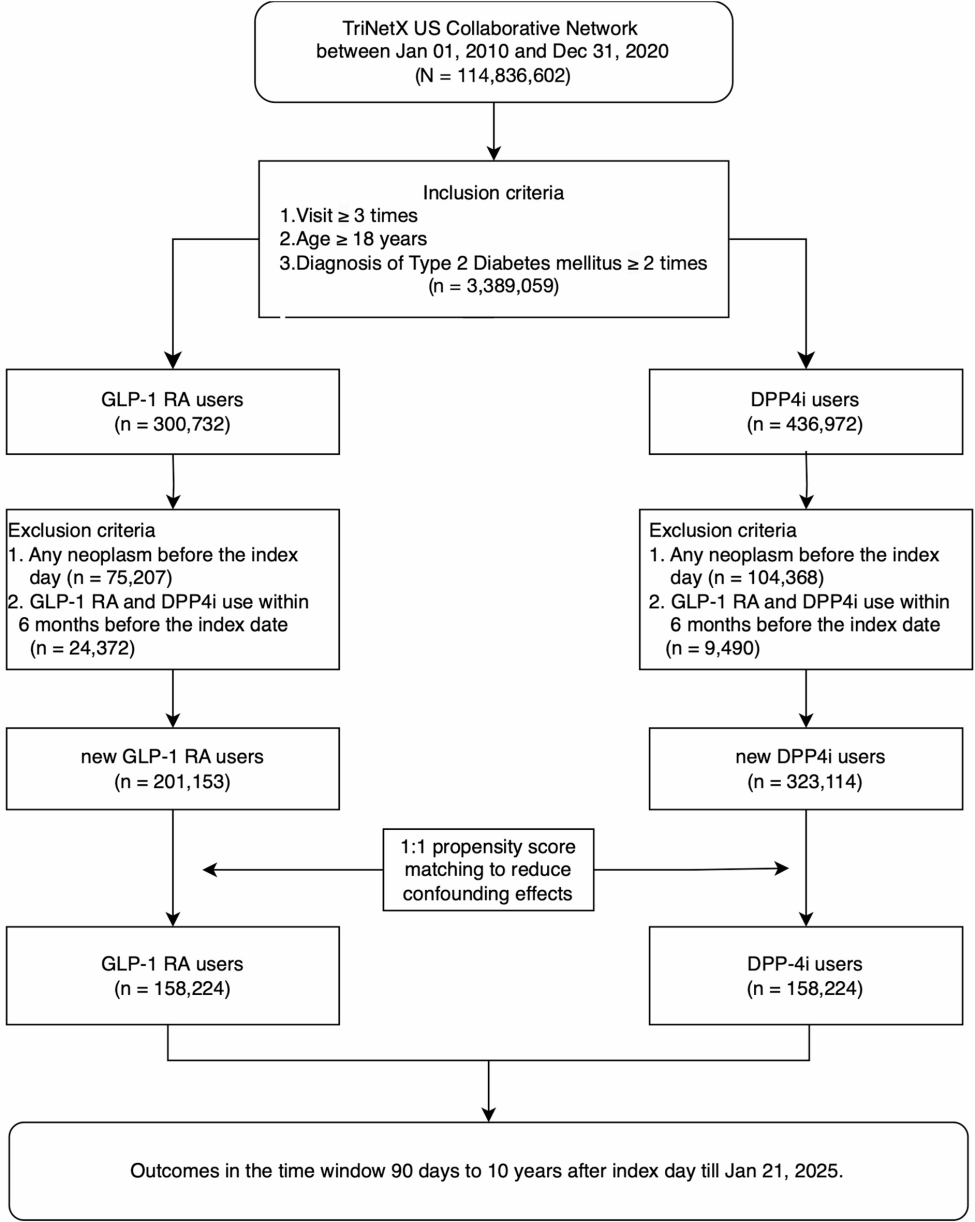
Patient enrollment. *GLP-1 RA* glucagon-like peptide-1 receptor agonist, *DPP4i* dipeptidyl peptidase-4 inhibitor.

This retrospective population-based cohort study used both new-user and active comparator designs to mitigate biases in observational studies and enhance validity by approximating randomized controlled trial (RCT) conditions^23^. Dipeptidyl peptidase-4 inhibitors (DPP4is) were chosen as the comparator because they share an incretin-based mechanism with GLP-1 RAs and are used as second-line anti-diabetic agents^24^. From the TriNetX platform, we obtained patient data (more than 114 million records). The present study focused on individuals aged ≥ 18 years with at least three clinical visits and a confirmed diagnosis of T2DM recorded at least twice (*n* = 3,389,059) from January 1, 2005, to December 31, 2020. The cohort was divided into two treatment groups: users of GLP-1 RAs (*n* = 300,732) and users of DPP4is (*n* = 436,972). Participants with prior neoplasms or recent (6-month) use of either drug class were excluded, resulting in 201,153 new GLP-1 RA users and 323,114 new DPP4i users (Fig. 1).

GLP-1 RA exposure was identified using the Anatomical Therapeutic Chemical (ATC) code A10BJ, which includes exenatide, liraglutide, lixisenatide, albiglutide, dulaglutide, and semaglutide. DPP-4 inhibitor exposure was identified using ATC code A10BH, which includes sitagliptin, vildagliptin, saxagliptin, linagliptin, and alogliptin.

The first medication exposure was defined as the index event, with a 3-month lag applied to all exposures to minimize protopathic bias and allow for sufficient latency after cohort entry. The outcomes of interest were evaluated within 90 days to 10 years after the index date, with follow-up extending until January 21, 2025. This study adhered to the Strengthening the Reporting of Observational Studies in Epidemiology reporting guidelines for observational studies^25^. Cohort definitions in TriNetX are detailed in eMethod 1 of the supplementary file.

### Propensity score matching

Propensity score matching (PSM) was performed to compare new GLP-1 RA users with new DPP4i users. PSM was performed in a 1:1 ratio to mitigate the effects of potential confounding factors. The PSM full model was adjusted for pre-specified baseline covariates, including demographic factors (e.g., proportion of male patients and ethnic composition of the study cohort), comorbidities, medication use, and laboratory metrics (details are shown in Table 1). Diagnostic codes for baseline variables are listed in Supplementary Materials (eMethod 1 of supplementary file).

**Table 1:** Baseline patient characteristics before and after propensity score matching.

Variables	Before matching			After matching		
	GLP-1 RA (*n* = 182,915)	DPP4i (*n* = 297,490)	SMD^#^	GLP-1 RA (*n* = 158,224)	DPP4i (*n* = 158,224)	SMD^#^
Age at Index (years), Mean ± SD	55.4 ± 12.2	60.3 ± 12.1	0.398	56.7 ± 11.7	56.4 ± 12.3	0.023
Male, n (%)	78,757 (43.1)	145,442 (48.9)	0.117	70,379 (44.5)	70,470 (44.5)	0.001
*Race, n (%)*						
White	109,518 (59.9)	168,710 (56.7)	0.064	94,715 (59.9)	96,109 (60.7)	0.018
African American	29,415 (16.1)	48,819 (16.4)	0.009	25,336 (16.0)	25,268 (16.0)	0.001
Asian	5723 (3.1)	18,951 (6.4)	0.153	5459 (3.5)	4937 (3.1)	0.019
Other	5263 (2.9)	9945 (3.3)	0.027	4634 (2.9)	4552 (2.9)	0.003
Unknown	30,741 (16.8)	46,529 (15.6)	0.032	26,056 (16.5)	25,258 (16.0)	0.014
*Ethnicity, n (%)*						
Hispanic or Latino	17,879 (9.8)	30,816 (10.4)	0.019	15,534 (9.8)	15,930 (10.1)	0.008
Not Hispanic or Latino	112,108 (61.3)	185,617 (62.4)	0.023	97,292 (61.5)	98,138 (62)	0.011
Unknown Ethnicity	52,928 (28.9)	81,057 (27.2)	0.038	45,398 (28.7)	44,156 (27.9)	0.017
*Lifestyles, n (%)*						
Problems related to lifestyle	6019 (3.3)	7279 (2.4)	0.051	4579 (2.9)	4495 (2.8)	0.003
Tobacco use	4722 (2.6)	6377 (2.1)	0.029	3838 (2.4)	3778 (2.4)	0.002
Alcohol related disorders	1894 (1)	3991 (1.3)	0.028	1699 (1.1)	1723 (1.1)	0.001
Nicotine dependence	12,523 (6.8)	19,934 (6.7)	0.006	10,673 (6.7)	10,658 (6.7)	< 0.001
*Socioeconomic status, n (%)*						
Persons with potential health hazards related to socioeconomic and psychosocial circumstances	2115 (1.2)	2942 (1)	0.016	1680 (1.1)	1680 (1.1)	< 0.001
Problems related to employment						
and unemployment	219 (0.1)	314 (0.1)	0.004	176 (0.1)	212 (0.1)	0.007
Problems related to housing and						
economic circumstances	679 (0.4)	1153 (0.4)	0.003	580 (0.4)	571 (0.4)	0.001
*History of cancer, n (%)*						
Family history of primary malignant neoplasm	4219 (2.3)	4935 (1.7)	0.046	3276 (2.1)	3253 (2.1)	0.001
Personal history of malignant neoplasm	1659 (0.9)	3495 (1.2)	0.026	1500 (0.9)	1470 (0.9)	0.002
Genetic susceptibility to malignant neoplasm	51 (0.0)	38 (0.0)	0.011	41 (0.0)	31 (0.0)	0.004
*Comorbidity, n (%)*						
Asthma	14,585 (8)	16,748 (5.6)	0.093	11,127 (7)	11,138 (7)	< 0.001
COPD	7426 (4.1)	14,408 (4.8)	0.038	6598 (4.2)	6643 (4.2)	0.001
Respiratory failure history	3101 (1.7)	7444 (2.5)	0.056	2836 (1.8)	2888 (1.8)	0.002
Other interstitial pulmonary diseases	876 (0.5)	1735 (0.6)	0.014	765 (0.5)	751 (0.5)	0.001
Pulmonary fibrosis, unspecified	479 (0.3)	1148 (0.4)	0.022	428 (0.3)	452 (0.3)	0.003
Hypertensive diseases	98,247 (53.7)	151,692 (51)	0.055	82,195 (51.9)	81,795 (51.7)	0.005
Heart failure	11,017 (6)	21,911 (7.4)	0.054	9758 (6.2)	9727 (6.1)	0.001
Ischemic heart diseases	23,525 (12.9)	43,942 (14.8)	0.055	20,681 (13.1)	20,593 (13.0)	0.002
Dyslipidemia	90,218 (49.3)	132,204 (44.4)	0.098	74,274 (46.9)	74,039 (46.8)	0.003
Overweight and obesity	57,050 (31.2)	52,001 (17.5)	0.324	41,576 (26.3)	41,013 (25.9)	0.008
Cerebrovascular diseases	8038 (4.4)	18,072 (6.1)	0.076	7331 (4.6)	7309 (4.6)	0.001
Diseases of arteries, arterioles and capillaries	10,202 (5.6)	19,806 (6.7)	0.045	8940 (5.7)	8928 (5.6)	< 0.001
Chronic kidney disease	15,547 (8.5)	32,209 (10.8)	0.079	13,942 (8.8)	13,815 (8.7)	0.003
Transplanted organ	1715 (0.9)	3980 (1.3)	0.038	1571 (1.0)	1569 (1.0)	< 0.001
*Medication, n (%)*						
Insulin	66,023 (36.1)	73,485 (24.7)	0.250	48,675 (30.8)	47,768 (30.2)	0.012
Biguanides	79,312 (43.4)	101,964 (34.3)	0.187	62,986 (39.8)	62,616 (39.6)	0.005
SGLT2i	17,084 (9.3)	9535 (3.2)	0.255	9566 (6.0)	8854 (5.6)	0.019
Sulfonylureas	35,752 (19.5)	60,058 (20.2)	0.016	30,865 (19.5)	30,856 (19.5)	< 0.001
Thiazolidinediones	8036 (4.4)	12,682 (4.3)	0.006	6845 (4.3)	6888 (4.4)	0.001
Alpha glucosidase inhibitors	402 (0.2)	628 (0.2)	0.002	344 (0.2)	341 (0.2)	< 0.001
Other blood glucose lowering drugs	1704 (0.9)	2563 (0.9)	0.007	1363 (0.9)	1385 (0.9)	0.001
Lipid modifying agents	78,515 (42.9)	115,970 (39)	0.080	64,672 (40.9)	64,466 (40.7)	0.003
ACEI	48,780 (26.7)	72,466 (24.4)	0.053	40,307 (25.5)	40,096 (25.3)	0.003
ARBs	29,045 (15.9)	39,343 (13.2)	0.075	23,409 (14.8)	23,318 (14.7)	0.002
Beta-blocker	43,123 (23.6)	74,487 (25)	0.034	37,115 (23.5)	37,022 (23.4)	0.001
Calcium channel blockers	30,067 (16.4)	50,960 (17.1)	0.019	25,630 (16.2)	25,375 (16.0)	0.004
Diuretics	48,202 (26.4)	72,774 (24.5)	0.043	40,153 (25.4)	39,861 (25.2)	0.004
Hypnotics and sedatives	26,779 (14.6)	43,606 (14.7)	0.001	22,517 (14.2)	22,484 (14.2)	0.001
NSAID	17,343 (9.5)	21,112 (7.1)	0.087	13,544 (8.6)	13,378 (8.5)	0.004
*Laboratory, mean ± SD*						
Hemoglobin A1c (%)	8.6 ± 2.3	8.2 ± 2.1	0.181	8.6 ± 2.3	8.4 ± 2.2	0.053
BMI(kg/m^2^)	36.8 ± 8.2	33.0 ± 7.7	0.485	36.1 ± 8.0	35.5 ± 7.9	0.074
Albumin (g/dL)	4.1 ± 0.5	4.0 ± 0.6	0.145	4.0 ± 0.5	4.0 ± 0.5	0.043
Hemoglobin (g/dL)	13.4 ± 1.9	12.9 ± 2.1	0.213	13.3 ± 1.9	13.2 ± 2.0	0.067
eGFR (mL/min/1.73m^2^)	85.0 ± 26.2	78.0 ± 28.8	0.253	83.1 ± 26.0	83.5 ± 28.1	0.015
Sodium(mmol/L)	138.2 ± 3.0	138.1 ± 3.3	0.035	138.3 ± 3.1	138.0 ± 3.2	0.078
UACR (mg/g)	483.0 ± 10968.1	486.0 ± 11808.7	< 0.001	413.9 ± 7137.6	525.7 ± 14007.4	0.010
LDL (mg/dL)	91.7 ± 38.7	91.4 ± 39.2	0.006	90.9 ± 38.7	93.6 ± 39.4	0.068
Triglyceride (mg/dL)	198.4 ± 208.8	184 ± 191.2	0.072	195.3 ± 201.7	194.4 ± 211.3	0.004
ALT (U/L)	31.7 ± 44.4	31.1 ± 45.6	0.012	31.4 ± 46.9	32.3 ± 40.5	0.021
AST (U/L)	26.0 ± 53.3	27.1 ± 48.9	0.021	26.0 ± 57.7	27 ± 39.4	0.019
Alkaline phosphatase (U/L)	85.6 ± 35.5	85.3 ± 45.3	0.007	85.5 ± 36.1	85.2 ± 42.3	0.010

The propensity score matching includes all parameters in this table to achieve balanced cohorts. *GLP-1 RA* glucagon-like peptide-1 receptor agonist, *DDP4i* dipeptidyl peptidase-4 inhibitor, *SMD* standardized mean difference, *SD* standard deviation, *COPD* chronic obstructive pulmonary disease, *SGLT2i* sodium-glucose cotransporter 2 inhibitor, *ACEI* angiotensin-converting enzyme inhibitor, *ARBs* angiotensin II receptor blockers, *NSAID* nonsteroidal anti-inflammatory drug, *BMI* body mass index, *eGFR* estimated glomerular filtration Rate, *UACR* urine albumin/creatinine ratio, *LDL* low density lipoprotein, *ALT* alanine aminotransferase, *AST* aspartate aminotransferase. ^#^A SMD value below 0.1 indicates a negligible difference between groups.

We used the PSM tool integrated within TriNetX to compute propensity scores and perform 1:1 matching. Propensity scores were generated through logistic regression, and matching was performed with a greedy nearest-neighbor algorithm, applying a caliper of 0.1 for pooled standardized mean differences (SMDs). The analysis was performed using Python (Python Software Foundation, Wilmington, DE, USA) and R (version 3.4.4; R Foundation for Statistical Computing, Vienna, Austria). This approach ensured the creation of comparable groups, thereby enhancing the accuracy of treatment effect analyses (eMethod 2 of supplementary file).

### Study outcomes

We evaluated several clinical outcomes related to lung health in patients treated with GLP-1 RAs and DPP4is. The primary outcome was the incidence of lung cancer, with additional analyses examining specific tumor sites, such as the trachea and bronchus. The secondary outcomes included influenza and pneumonia, other acute lower lung infection, suppurative lung disease, and pulmonary fibrosis. We included a broader range of respiratory conditions as secondary outcomes to capture more clinically relevant morbidities potentially influenced by the exposure. These encompassed both upper and lower airway diseases of varying acuity, from acute infections to chronic inflammatory disorders. This approach allowed us to assess the consistency of associations across related phenotypes and explore possible mechanism-specific effects. These outcomes were evaluated to compare risks between the two treatment groups (eMethod 1 of supplementary file).

### Sensitivity analyses

To test the robustness of our findings, we performed analyses using both positive and negative outcome controls. GLP-1 RAs have been demonstrated to reduce cardiovascular and renal risks in patients with T2DM^26,27^. Therefore, major adverse cardiovascular events (e.g., cerebral infarction, myocardial infarction, and death) and major adverse kidney events (e.g., acute and advanced kidney failure, dialysis initiation, and death) were used as positive outcome controls. By contrast, bone fractures and scleroderma, which have no reported associations with GLP-1 RAs, were included as negative outcome controls.

To validate our findings, we performed sensitivity analyses: (1) by extending the time lag for outcome analysis from 3 to 6 months and 12 months after the index date, (2) by using different PSM models adjusted for multiple confounders (PSM Model 1 was matched for age, sex, race, body mass index [BMI], lifestyle, and socioeconomic status; Model 2 was matched for baseline comorbidities in addition to Model 1 covariates; and Model 3 was further matched for baseline medication use), and (3) by analyzing the risk profile across different time frames (3 months to 3 years, 3 months to 5 years, and 3 months to 7 years).

### Statistical analysis

The baseline characteristics of the treatment groups were analyzed both before and after PSM. Summary statistics for continuous variables are presented as mean ± standard deviation values, whereas those for categorical variables are presented as frequency (%) values. SMDs were calculated to determine the balance of baseline characteristics between the groups, with an SMD value of < 0.1 indicating minimal differences^28^.

Study outcomes were analyzed through risk and survival analyses. A Cox proportional-hazards model was used to calculate the hazard ratio (HR) and corresponding 95% confidence interval (CI) values for comparing outcome incidence between groups. Kaplan–Meier curves were generated to visualize time-to-event data, and the log-rank test was used to compare survival distributions. The proportional-hazards assumption was assessed using the generalized Schoenfeld approach integrated into the TriNetX platform through R’s Survival package (version 3.2-3). If this assumption was violated, HRs were calculated for different periods. We tested seven hypotheses with a false discovery rate of 0.05, correcting P values using the Benjamini–Hochberg procedure^29^.

Subgroup analyses were performed to examine the consistency of treatment effects across key baseline characteristics, such as age (≥ 65 and < 65 years), sex (male and female), BMI (≥ 30 and < 30 kg/m²), estimated glomerular filtration rate (eGFR; ≥60 and < 60 mL/min/1.73 m^2^), and HbA1c (≥ 7% and < 7%). Further analyses compared lung conditions between GLP-1 RAs and other antidiabetic medications, including insulin, biguanides, sodium-glucose cotransporter 2 inhibitor, sulfonylureas, and thiazolidinediones.

All statistical analyses were performed using the TriNetX real-time analytics platform. A two-sided *p* value of < 0.05 indicated statistical significance.

## Results

### Baseline characteristics of the study cohort

After PSM incorporating all the baseline parameters, each drug group had 158,224 users. The baseline characteristics of the treatment groups before and after PSM are summarized in Table 1. Before PSM, significant differences were observed between the groups. GLP-1 RA users were younger than DPP4i users (mean age: 55.4 vs. 60.3 years; SMD: 0.398). Furthermore, the prevalence of obesity and insulin use was higher among GLP-1 RA users than among DPP4i users (obesity, BMI ≥ 35 kg/m²: 26.8% vs. 16.6% [SMD: 0.249]; insulin use: 36.1% vs. 24.7% [SMD: 0.250]). After PSM, these differences were minimized, with GLP-1 RA and DPP4i users having similar mean age (56.7 vs. 56.4 years; SMD = 0.023) and BMI (23.2% vs. 23.1%; SMD = 0.003) observed between the groups. Moreover, both groups exhibited similar sex distributions (44.5% men) and racial compositions. Comorbidities such as hypertension, dyslipidemia, and chronic kidney disease were prevalent in both groups, with minimal differences noted after PSM (e.g., hypertension: 51.9% vs. 51.7% [SMD: 0.005]). After PSM, between-group balance was observed also in laboratory results such as HbA1c (8.6% vs. 8.4%; SMD: 0.053) and eGFR (83.1 vs. 83.5 mL/min/1.73 m²; SMD: 0.015). Lifestyle factors such as nicotine dependence and alcohol-related disorders exhibited negligible differences between the groups after PSM. Medication use patterns (biguanides, sulfonylureas, and lipid-modifying agents) were similar between the two groups after PSM. Overall, PSM effectively reduced baseline differences between GLP-1 RA and DPP4i users, ensuring similarity for subsequent analyses.

### Lung cancer risk in GLP-1 RA versus DPP4i users

The median follow-up was 1882 days (interquartile range 1153) for GLP-1 RA users and 2078 days (interquartile range 1682) for DPP4i user. Event-free survival rates of lung cancer differed significantly between GLP-1 RA and DPP4i users (*p* < 0.001; eFigure 1 of supplementary file). At 5-year follow-up, event-free survivals rate of lung cancer were approximately 99.4% and 99.2% for GLP-1 RA and DPP4i users, respectively. By 10 years, these rates were approximately 98.6% and 98.3% for GLP-1 RA and DPP4i users, respectively.

After PSM, GLP-1 RA users had a significantly lower risk of lung cancer than did DPP4i users (HR 0.86; 95% CI 0.80–0.94; Table 2). The risk reduction remained significant specifically for bronchial cancers (HR 0.91; 95% CI 0.84–0.99). These findings remained significant after Benjamini-Hochberg false discovery rate correction.

**Table 2:** Pulmonary outcomes in type 2 diabetes.

Pulmonary outcomes	After propensity score matching with a longest 10-year follow-up				
	GLP-1 RA (*n* = 158,224)	DDP4i (*n* = 158,224)	GLP-1 RA vs. DDP4i		
	Events/ number at risk^†^ (%)		HR (95%CI)	*p* value	FDR-adjusted *p* value
*Primary outcomes*					
Lung cancer	1083/158,224 (0.7)	1396/158,224 (0.9)	0.82 (0.76–0.89)	< 0.001	< 0.001
Trachea	10/158,224 (< 0.1)	14/158,224 (< 0.1)	0.47 (0.18–1.21)	0.109	0.191
Bronchus	1079/158,224 (0.7)	1389/158,224 (0.9)	0.87 (0.81–0.95)	0.001	0.001
*Secondary outcomes*					
Influenza and pneumonia	16,611/146,544 (11.3)	19,444/146,248 (13.3)	0.94 (0.92–0.96)	< 0.001	< 0.001
Other acute lower lung infection	8318/147,667 (5.6)	10,396/146,602 (7.1)	0.85 (0.82–0.87)	< 0.001^‡^	< 0.001
Suppurative lung disease	392/157,816 (0.2)	586/157.781 (0.4)	0.74 (0.65–0.84)	< 0.001	< 0.001
Pulmonary fibrosis	1792/157,266 (1.1)	2192/157,256 (1.4)	0.92 (0.87–0.98)	0.010	0.018

*GLP-1 RA* glucagon-like peptide-1 receptor agonist, *DPP4i* dipeptidyl peptidase-4 inhibitor, *HR* hazard ratio, *CI* confidence interval, *FDR* Benjamini-Hochberg false discovery rate. ^†^ The number at risk refers to patients who have not experienced the outcome before follow-up begins. ^‡^ This indicates a violation of the proportional hazard’s assumption in Cox regression.

### Pulmonary infection and fibrosis risk in GLP-1 RA versus DPP4i users

GLP-1 RA users consistently show higher event-free rates for influenza, pneumonia (log rank *p* < 0.001), acute lower lung infection (log rank *p* < 0.001), suppurative lung disease (log rank *p* < 0.001), and pulmonary fibrosis (log rank *p* = 0.01), indicating better outcomes compared to DPP4i users (eFigure 2 of supplementary file). Table 2 shows that GLP-1 RA users is associated with significantly lower risks of secondary outcomes compared to DPP4i users. For influenza and pneumonia, the HR was 0.94 (95% CI 0.92–0.96); for other acute lower lung infections, HR was 0.85 (95% CI 0.82–0.87); for suppurative lung disease, HR was 0.74 (95% CI 0.65–0.84); and for pulmonary fibrosis, HR was 0.92 (95% CI 0.87–0.98).

### Results of subgroup analyses

**Fig. 2:**
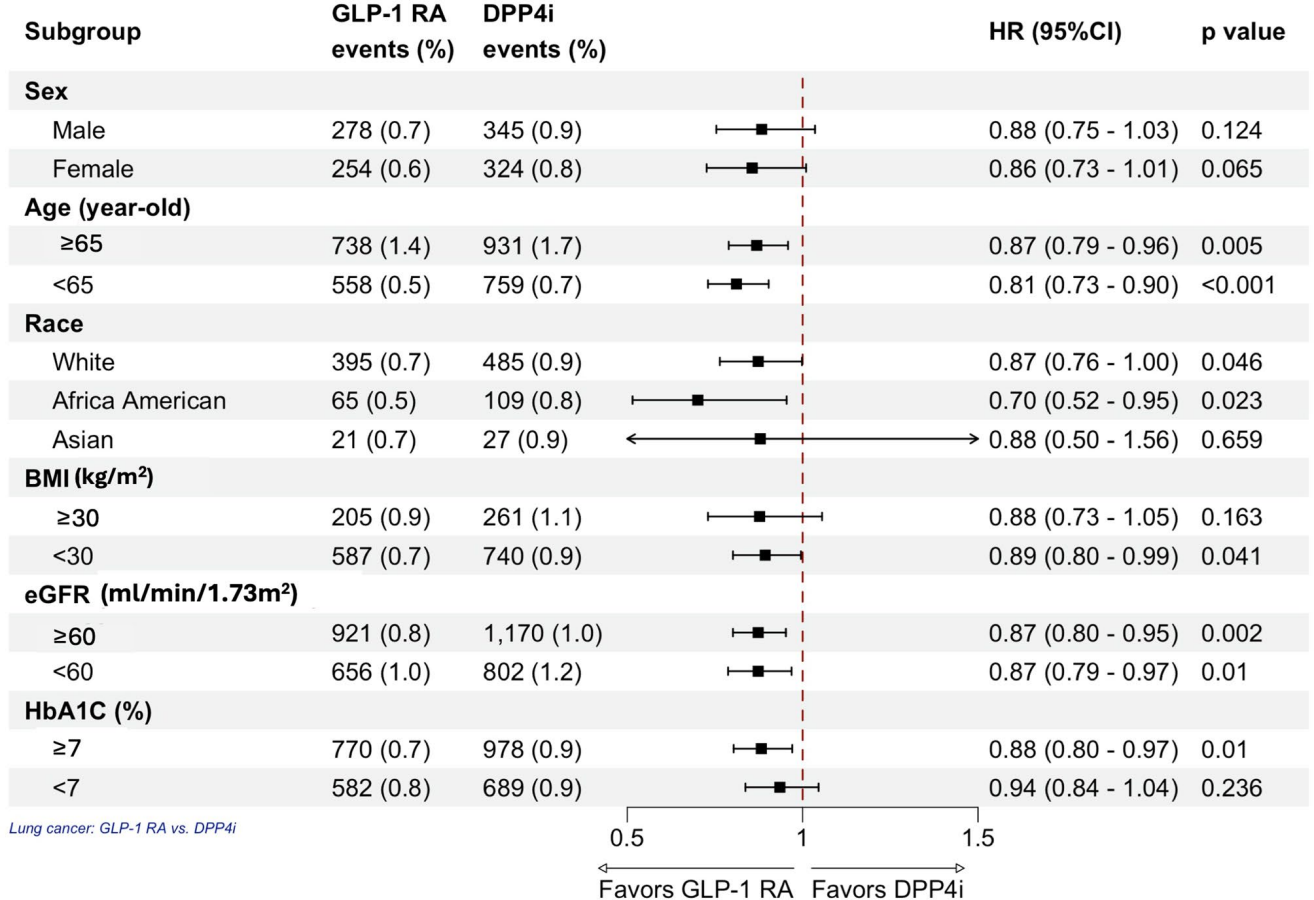
Subgroup analysis for lung cancer. Lung cancer risk in GLP-1 RA users versus DPP4i users. The vertical line indicates an HR of 1.00. A lower limit of the 95% CI greater than 1.00 indicates a significantly higher risk. *GLP1-RA* glucagon-like peptide-1 receptor agonist, *DPP4i* dipeptidyl peptidase-4 inhibitor, *HR* hazard ratio, *CI* confidence interval, *BMI* body mass index, *eGFR* estimated glomerular filtration rate, *HbA1c* glycated hemoglobin.

Subgroup analyses revealed that GLP-1 RAs use was associated with a reduced risk of lung cancer across multiple demographic groups (Fig. 2). The protective effect was observed in patients aged < 65 years as well as in those aged ≥ 65 years. The risk reduction was also significant among African American patients and White patients. Similar protective effects were observed across various eGFR groups and in individuals with BMI < 30 Kg/m^2^ and HbA1c levels of ≥ 7%.

**Fig. 3:**
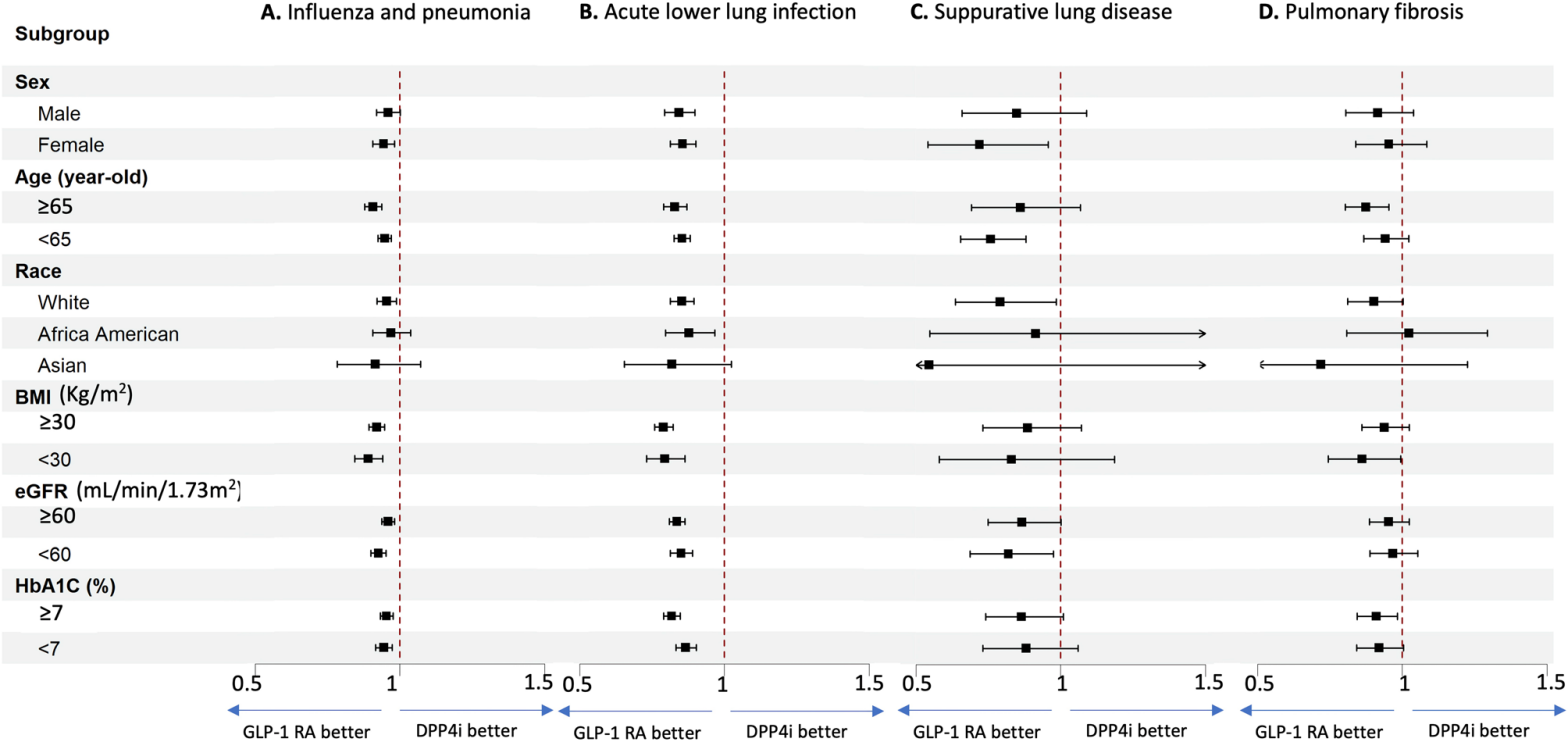
Subgroup analysis for lung infection and fibrosis. Risk of other lung conditions includes (A) influenza and pneumonia, (B) acute lower lung infection, (C) suppurative lung disease, and (D) pulmonary fibrosis in GLP-1 RA users versus DPP4i users. The vertical line indicates an HR of 1.00. A lower limit of the 95% CI greater than 1.00 indicates a significantly higher risk. *GLP1-RA* glucagon-like peptide-1 receptor agonist, *DPP4i* dipeptidyl peptidase-4 inhibitor, *HR* hazard ratio, *CI* confidence interval, *BMI* body mass index, *eGFR* estimated glomerular filtration rate, *HbA1c* glycated hemoglobin.

For secondary pulmonary outcomes comparing GLP‑1 RA and DPP4i treatments across various groups. Across nearly all subgroups, GLP‑1 RA is consistently associated with a reduced risk of influenza and pneumonia, acute lower lung infection, suppurative lung disease, and pulmonary fibrosis, with effect sizes generally favoring GLP‑1 RA over DPP4i (Fig. 3). These protective effects remain robust regardless of demographic or clinical factors, highlighting that GLP-1 RA use is associated with reduced risk of respiratory infection and lung fibrosis across diverse patient populations with T2DM (detail are shown in eFigs. 3, 4, 5 and 6 of supplementary file).

### Results of comparison of GLP-1 RA with other antidiabetic medications

**Fig. 4:**
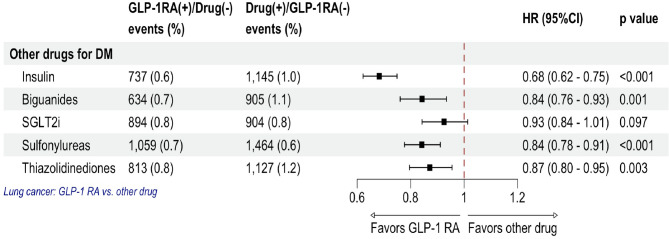
Lung cancer risk in patients using GLP-1 RAs versus those using other antidiabetic medications. The vertical line indicates an HR of 1.00. A lower 95% CI limit of > 1.00 indicates a markedly higher risk. *GLP1-RA* glucagon-like peptide-1 receptor agonist, *SGLT2i* sodium–glucose cotransporter 2 inhibitor, *HR* hazard ratio, *CI* confidence interval.

Figure 4 shows the risk comparison of lung cancer between GLP-1 RA and other antidiabetic medications. The users of GLP-1 RAs consistently had a lower risk of lung cancer than did the users of most other diabetes medications, such as insulin (HR 0.68; 95% CI 0.62–0.75), biguanides (HR 0.84; 95% CI 0.76–0.93), sulfonylureas (HR 0.84; 95% CI 0.78–0.91), and thiazolidinediones (HR 0.87; 95% CI 0.80–0.95). However, the risk was similar between GLP-1 RA users and sodium–glucose cotransporter 2 inhibitor (SGLT2i) users (HR 0.93; 95% CI 0.84–1.01).

**Fig. 5:**
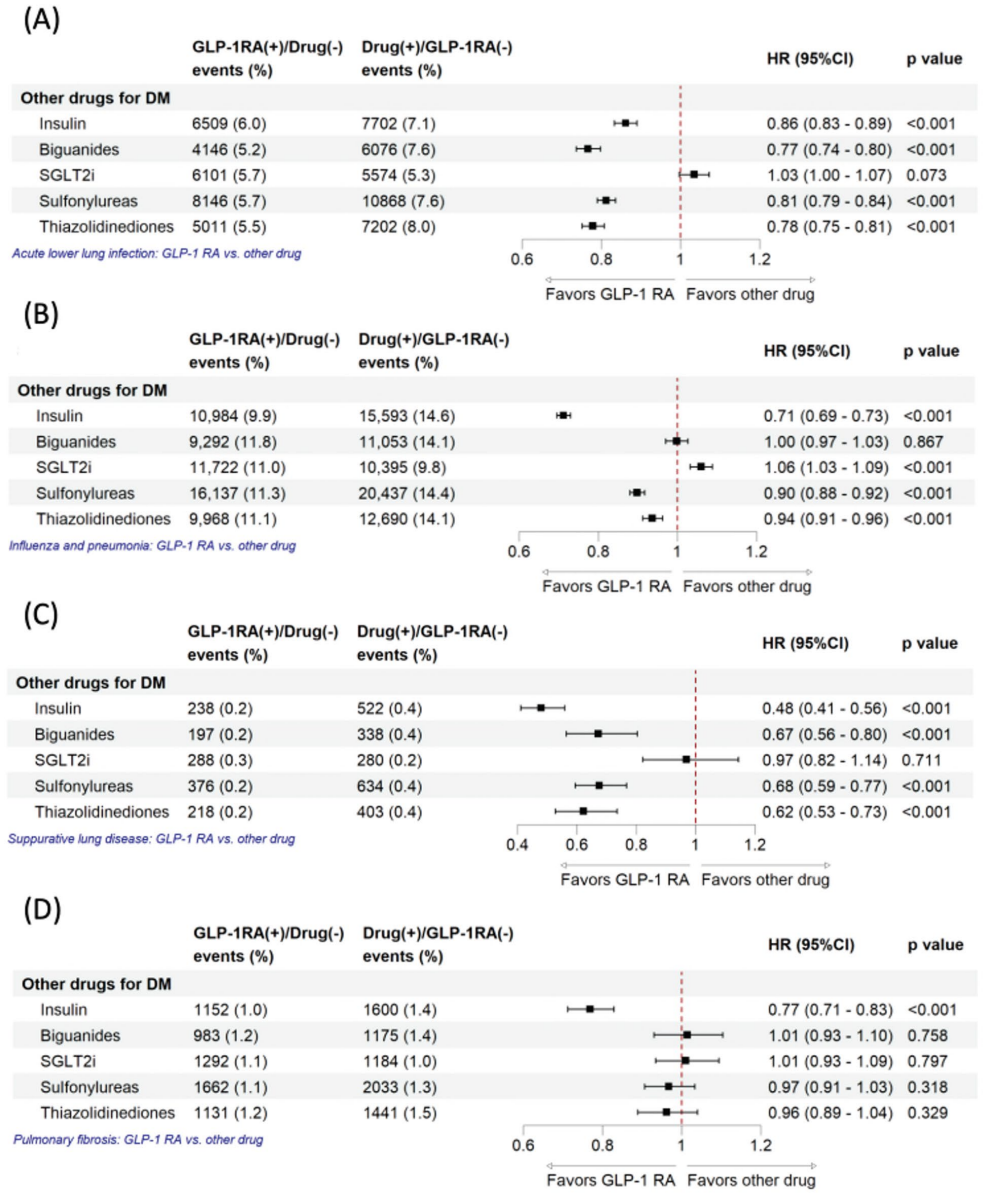
Risk of other lung conditions in patients using GLP-1 RAs versus other antidiabetic medications. Risk of other lung conditions includes **A** influenza and pneumonia, **B** acute lower lung infection, **C** suppurative lung disease, and **D** pulmonary fibrosis. The vertical line indicates an HR of 1.00. A lower 95% CI limit of > 1.00 indicates a markedly higher risk. *GLP1-RA* glucagon-like peptide-1 receptor agonist, *SGLT2i* sodium–glucose cotransporter 2 inhibitor, *HR* hazard ratio, *CI* confidence interval.

Figure 5 shows that GLP-1 RA users had lower event rates and HRs for acute lower lung infections and suppurative lung disease versus most other diabetes drugs, especially insulin, biguanides, sulfonylureas, and thiazolidinediones, but not always SGLT2i. For influenza, pneumonia, and pulmonary fibrosis, GLP-1 RA showed benefits mainly over insulin. Overall, GLP-1 RA generally lowers pulmonary risks compared to most drugs except SGLT2i.

### Results of sensitivity analyses

Positive outcome control analyses indicated that GLP-1 RA users had markedly lower risks of major adverse cardiovascular and kidney events than did DPP4i users. However, negative outcome control analyses revealed no significant between-group differences in the incidence of bone fractures or scleroderma (Supplementary Materials, eTable 1). Our findings remained consistent when the analysis time lag was extended from 3 to 6 or 12 months after the index date. However, pulmonary fibrosis lost significance with a 12-month lag (Supplementary Materials, eTable 2 and eTable 3). Furthermore, the results were consistent across various PSM models after adjustments were made for multiple confounders (Supplementary Materials, eTable 4) and across diverse temporal frameworks (Supplementary Materials, eTable 5).

## Discussion

Our large-scale, population-based study examined the association between GLP-1 RAs and pulmonary illness in patients with T2DM, utilizing real-world data (EHR data) from the TriNetX US Collaborative Network. The results indicated a significantly 14% lower risk of lung cancer among GLP-1 RA users compared to DPP4i users. Additionally, there was a 6–26% reduction in the risk of lung infection and an 8% reduction in pulmonary fibrosis in the group of GLP-1 RA users. Sensitivity analyses confirmed the robustness of these findings, supporting the notion that GLP-1 RAs may provide a protective benefit on lung health. Compared to other anti-diabetic medications, GLP-1 RAs had a 13–32% lower risk of lung cancer. These results strengthen the case for GLP-1 RAs as therapies beyond glycemic control and support further research into their mechanisms and effects on lung health in diabetes.

GLP-1 RAs have been studied for their effects on lung cancer. Current evidence indicates no significant association between GLP-1 RAs and lung cancer risk^13^. A Mendelian randomization study and a systematic review of RCTs also found no evidence linking GLP-1 RAs to lung cancer^13^. However, a TriNetX study found that GLP-1 RA use was associated with a reduced risk of respiratory cancer (HR 0.65; 95% CI 0.55–0.77) compared to non-users among patients with obesity over a 5-year follow-up^14^. Another TriNetX study found that patients on noninsulin antidiabetic drugs (excluding alpha-glucosidase inhibitors) had a lower risk of lung cancer than those using insulin, with GLP-1 RAs being the most effective in reducing this risk (HR 0.49; 95% CI 0.41–0.59) in T2DM patients within a longest of 15 years observation^15^. In our present study, we employed a new-user, active-comparator design using the same dataset of TriNetX. Our findings provide additional evidence to suggest that GLP-1 RA therapy may play a significant role in reducing the risk of lung cancer in clinical practice. While these results contribute to the ongoing debate, further research with rigorous design is warranted to confirm these observations and fully elucidate the underlying mechanisms.

Our study also shows that GLP-1 RAs have a greater ability to reduce the risk of other lung diseases, such as acute lower respiratory infections, suppurative lung disease, and pulmonary fibrosis. Previous studies have demonstrated that GLP-1 RAs can reduce pulmonary fibrosis by downregulating collagen synthesis, restoring ACE2 expression to rebalance the RAS pathway, and increasing surfactant proteins to support alveolar structure and lung function in animal models^30^ which is consistent with our real-world findings. Other studies have reported similar results, showing that GLP-1 RAs can lower the risk of lung infections such as pneumonia and bronchitis^31^. However, the exact mechanisms by which GLP-1 RAs reduce the incidence of lung infections remain unclear.

The precise mechanism through which GLP-1 RAs reduce respiratory illness risk remains unclear, but several potential explanations have been proposed. A key reason is the well-documented anti-inflammatory and antioxidant properties of GLP-1 RAs^20,32^. These drugs can regulate the activity of immune cells, suppress the activation of the nuclear factor-κB pathway, and reduce the production of proinflammatory cytokines^33^. In addition, they induce changes in inflammatory and oxidative stress biomarkers^34^ contributing to improved disease management. GLP-1-based therapies, including GLP-1 RAs and DPP4is, exert anti-inflammatory effects on various organs, such as the lungs, liver, brain, and kidneys; therefore, these drugs hold promise for treating inflammation-related diseases^21,35^. The relatively low effectiveness of DPP4is in mitigating lung cancer risk may be attributable to their potential to increase substance P ^36^, which is associated with cancer development^37^. This mechanism may explain why DPP4i users have a higher risk of lung cancer than do GLP-1 RA users.

The present study has some limitations. First, Due to its retrospective design, this study is subject to inherent biases, and causality cannot be established. Key factors in lung cancer development—such as smoking (pack-years, duration, or changes over time), occupational exposure, air pollution, and family history^38,39^—were unavailable, and differences in these variables among patients may have affected our results. Although we accounted for many baseline conditions, certain factors might not have been documented in the TriNetX platform and thus could not be included. Second, our reliance on data from the TriNetX platform might have introduced coding-related inaccuracies. Information on patient compliance and actual medication use was not available, which might have affected the assessment of treatment effects. Third, we did not categorize lung cancer into specific subtypes such as adenocarcinoma, squamous cell carcinoma, and small cell carcinoma. This limited our ability to evaluate potential variations in risk among different histological types of lung cancer. Fourth, the reliability of cancer diagnoses in TriNetX, like other large real-world data sources, relies on the accuracy and completeness of EHRs from participating health systems. Nonetheless, studies in EHR data generally report high positive predictive values (over 80–90%) for the cancer diagnosis^40,41^. Moreover, we excluded individuals with prior cancer to ensure active follow-up, but acknowledge that cases diagnosed outside the network may be missed, potentially introducing ascertainment bias. Fifth, this study predominantly involved white people (about 60%). As a result, the applicability and generalizability of these findings to individuals from other ethnic backgrounds may be limited, since the results are based primarily on data from white participants. The finally, although this study considered contributing factors, the exact mechanism by which GLP-1 RAs affect lung health remains unclear. Future research should investigate how GLP-1 RAs reduce lung cancer and infection risk.

## Conclusion

Our findings indicate that GLP-1 RAs significantly lower the risk of lung cancer and may improve lung health, reinforcing their protective effects on pulmonary disease beyond glycemic control. Future research should also address unresolved confounders and evaluate the effectiveness of GLP-1 RAs in specific populations and across different lung disease subtypes.

## Supplementary Information

Below is the link to the electronic supplementary material.

Supplementary Material 1

## Data Availability

TriNetX is a comprehensive network that links together a diverse range of research centers, enabling collaboration and data sharing across multiple institutions. Through this platform, users are granted immediate access to anonymized data that is derived from the electronic health records maintained by various participating health-care organizations. This integration allows researchers to work with a vast and diverse dataset in real time. Access to these datasets and research tools is available to authorized researchers via the online portal at https://live.trinetx.com, providing a convenient and efficient way to support health research and analysis.
