# Supplementary material for: Association between glucagon-like peptide-1 receptor agonist therapy and respiratory illness in patients with type 2 diabetes: a retrospective observational cohort study
Source: Sci Rep. 2025 Oct 13;15:35625. doi: 10.1038/s41598-025-19657-5 (PMC12518849; doi:10.1038/s41598-025-19657-5)
Supplement: Supplementary file 1 — Supplementary Material 1 [file 41598_2025_19657_MOESM1_ESM.docx]

**Supplementary Online Content**

**eMethod 1** Code for cohorts, outcomes, and baseline data

**eMethod 2** The detail of propensity score matching in TriNetX platform

**eFigure1.** Kaplan–Meier plots of lung cancer

**eFigure2.** Kaplan–Meier plots of pulmonary infection and fibrosis

**eFigure 3.** Subgroup analysis for influenza and pneumonia

**eFigure 4**. Subgroup analysis for acute lower lung infection

**eFigure 5**. Subgroup analysis for suppurative lung disease

**eFigure 6**. Subgroup analysis for pulmonary fibrosis

**eTable 1.** The positive and negative outcomes control

**eTable 2.** Sensitivity test by extending the index date by 6 months

**eTable 3.** Sensitivity test by extending the index date by 12 months

**eTable 4.** PSM Models for confounding adjusting

**eTable 5.** Segmenting the follow-up into four periods (3 months to 3-, 5-, and 7-years post-index date)

**eMethod 1.** Code for cohorts, outcomes, and baseline data

1. **Cohorts definition**

### **Query Criteria for Cohort 1 (query name: GLP1_RA)**

|  | | | | | |
| --- | --- | --- | --- | --- | --- |
| Group 1 | | | | | |
|  | **Visit** | | | | |
|  | must have |  | visit | TNX:Visit | Visit |
|  | number of instances | | Greater than or equal to 3 instances | | |
|  | date constraint | | The terms in this group occurred at any time | | |
| Group 2 | | | | | |
|  | **DM** | | | | |
|  | must have |  | diagnosis | UMLS:ICD10CM:E11 | Type 2 diabetes mellitus (at least 18 years old at event) |
|  | number of instances | | Greater than or equal to 2 instances | | |
|  | date constraint | | The terms in this group occurred between Jan 1, 2010 and Dec 31, 2020 | | |
| Group 3 | | | | | |
|  | **Group 3A GLP1-RA** | | | | |
|  | must have |  | medication | NLM:ATC:A10BJ | Glucagon-like peptide-1 (GLP-1) analogues |
|  | date constraint | | The terms in this group occurred between Jan 1, 2010 and Dec 31, 2020 | | |
|  | event relationship | | Any instance of exclude DPP4i occurred within 3 months on or after the first instance of GLP1-RA | | |
|  | **Group 3B exclude DPP4i** | | | | |
|  | cannot have |  | medication | NLM:ATC:A10BH | Dipeptidyl peptidase 4 (DPP-4) inhibitors |
| Group 4 | | | | | |
|  | **Group 4A GLP1-RA** | | | | |
|  | must have |  | medication | NLM:ATC:A10BJ | Glucagon-like peptide-1 (GLP-1) analogues |
|  | date constraint | | The terms in this group occurred between Jan 1, 2010 and Dec 31, 2020 | | |
|  | event relationship | | Any instance of exclude drug before 6M occurred within 6 months and 1 day before the first instance of GLP1-RA | | |
|  | **Group 4B exclude drug before 6M** | | | | |
|  | cannot have |  | medication | NLM:ATC:A10BH | Dipeptidyl peptidase 4 (DPP-4) inhibitors |
|  |  | or | medication | NLM:ATC:A10BJ | Glucagon-like peptide-1 (GLP-1) analogues |
| Group 5 | | | | | |
|  | **Group 5A GLP1-RA** | | | | |
|  | must have |  | medication | NLM:ATC:A10BJ | Glucagon-like peptide-1 (GLP-1) analogues |
|  | date constraint | | The terms in this group occurred between Jan 1, 2010 and Dec 31, 2020 | | |
|  | event relationship | | Any instance of exclude Oncology occurred at least 1 day before the first instance of GLP1-RA | | |
|  | **Group 5B exclude Oncology** | | | | |
|  | cannot have |  | diagnosis | UMLS:ICD10CM:C00-D49 | Neoplasms |

### **Query Criteria for Cohort 2 (query name: DPP4i)**

| Group 1 | | | | | |
| --- | --- | --- | --- | --- | --- |
|  | **Visit** | | | | |
|  | must have |  | visit | TNX:Visit | Visit |
|  | number of instances | | Greater than or equal to 3 instances | | |
|  | date constraint | | The terms in this group occurred at any time | | |
| Group 2 | | | | | |
|  | **DM** | | | | |
|  | must have |  | diagnosis | UMLS:ICD10CM:E11 | Type 2 diabetes mellitus (at least 18 years old at event) |
|  | number of instances | | Greater than or equal to 2 instances | | |
|  | date constraint | | The terms in this group occurred between Jan 1, 2010 and Dec 31, 2020 | | |
| Group 3 | | | | | |
|  | **Group 3A DPP4i** | | | | |
|  | must have |  | medication | NLM:ATC:A10BH | Dipeptidyl peptidase 4 (DPP-4) inhibitors |
|  | date constraint | | The terms in this group occurred between Jan 1, 2010 and Dec 31, 2020 | | |
|  | event relationship | | Any instance of exclude DPP4i occurred within 3 months on or after the first instance of GLP1-RA | | |
|  | **Group 3B exclude GLP-1 RA** | | | | |
|  | cannot have |  | medication | NLM:ATC:A10BJ | Glucagon-like peptide-1 (GLP-1) analogues |
| Group 4 | | | | | |
|  | **Group 4A DPP4i** | | | | |
|  | must have |  | medication | NLM:ATC:A10BH | Dipeptidyl peptidase 4 (DPP-4) inhibitors |
|  | date constraint | | The terms in this group occurred between Jan 1, 2010 and Dec 31, 2020 | | |
|  | event relationship | | Any instance of exclude drug before 6M occurred within 6 months and 1 day before the first instance of GLP1-RA | | |
|  | **Group 4B exclude drug before 6M** | | | | |
|  | cannot have |  | medication | NLM:ATC:A10BH | Dipeptidyl peptidase 4 (DPP-4) inhibitors |
|  |  | or | medication | NLM:ATC:A10BJ | Glucagon-like peptide-1 (GLP-1) analogues |
| Group 5 | | | | | |
|  | **Group 5A DPP4i** | | | | |
|  | must have |  | medication | NLM:ATC:A10BH | Dipeptidyl peptidase 4 (DPP-4) inhibitors |
|  | date constraint | | The terms in this group occurred between Jan 1, 2010 and Dec 31, 2020 | | |
|  | event relationship | | Any instance of exclude Oncology occurred at least 1 day before the first instance of GLP1-RA | | |
|  | **Group 5B exclude Oncology** | | | | |
|  | cannot have |  | diagnosis | UMLS:ICD10CM:C00-D49 | Neoplasms |

| Inclusion criteria | | | |
| --- | --- | --- | --- |
|  | **Type 2 DM and target medications** | | |
|  | diagnosis | UMLS:ICD10CM:E11 | Type 2 diabetes mellitus (at least 18 years old at event) |
|  | diagnosis | UMLS:ICD10CM:I21 | Acute myocardial infarction |
|  | **Visit** | | |
|  | visit | TNX:Visit | Visit > 3 |
|  | Target medications |  |  |
|  | medication | NLM:ATC:A10BJ | Glucagon-like peptide-1 (GLP-1) analogues |
|  | medication | NLM:ATC:A10BH | Dipeptidyl peptidase 4 (DPP-4) inhibitors |
| Exclusion criteria | | | |
|  | **Autoimmune diseases** | | |
|  | diagnosis | UMLS:ICD10CM:C00-D49 | Neoplasms |
|  |  | C00–C96 | malignant neoplasms |
|  |  | D00–D09 | in situ neoplasms |
|  |  | D10–D36 | benign neoplasms |
|  |  | D37–D48 | neoplasms of uncertain or unknown behavior |
|  |  | D49 | neoplasms of unspecified behavior |

### **Outcome Definitions**

| Lung cancer | | |
| --- | --- | --- |
| Diagnosis | UMLS:ICD10CM:C33 | Malignant neoplasm of trachea |
| Diagnosis | UMLS:ICD10CM:C34 | Malignant neoplasm of bronchus and lung |
| Malignant neoplasm of unspecified main bronchus | | |
| Diagnosis | UMLS:ICD10CM:C34.0 | Malignant neoplasm of main bronchus |
| Diagnosis | UMLS:ICD10CM:C34.00 | Malignant neoplasm of unspecified main bronchus |
| Diagnosis | UMLS:ICD10CM:C34.01 | Malignant neoplasm of right main bronchus |
| Diagnosis | UMLS:ICD10CM:C34.02 | Malignant neoplasm of left main bronchus |
| Malignant neoplasm of upper lobe | | |
| Diagnosis | UMLS:ICD10CM:C34.1 | Malignant neoplasm of upper lobe, bronchus or lung |
| Diagnosis | UMLS:ICD10CM:C34.10 | Malignant neoplasm of upper lobe, unspecified bronchus or lung |
| Diagnosis | UMLS:ICD10CM:C34.11 | Malignant neoplasm of upper lobe, right bronchus or lung |
| Diagnosis | UMLS:ICD10CM:C34.12 | Malignant neoplasm of upper lobe, left bronchus or lung |
| Malignant neoplasm of middle lobe, bronchus or lung | | |
| Diagnosis | UMLS:ICD10CM:C34.2 | Malignant neoplasm of middle lobe, bronchus or lung |
| Malignant neoplasm of lower lobe | | |
| Diagnosis | UMLS:ICD10CM:C34.3 | Malignant neoplasm of lower lobe, bronchus or lung |
| Diagnosis | UMLS:ICD10CM:C34.31 | Malignant neoplasm of lower lobe, right bronchus or lung |
| Diagnosis | UMLS:ICD10CM:C34.30 | Malignant neoplasm of lower lobe, unspecified bronchus or lung |
| Diagnosis | UMLS:ICD10CM:C34.32 | Malignant neoplasm of lower lobe, left bronchus or lung |
| C34.8 Malignant neoplasm of overlapping sites of unspecified bronchus and lung | | |
| Diagnosis | UMLS:ICD10CM:C34.8 | Malignant neoplasm of overlapping sites of bronchus and lung |
| Diagnosis | UMLS:ICD10CM:C34.80 | Malignant neoplasm of overlapping sites of unspecified bronchus and lung |
| Diagnosis | UMLS:ICD10CM:C34.81 | Malignant neoplasm of overlapping sites of right bronchus and lung |
| Diagnosis | UMLS:ICD10CM:C34.82 | Malignant neoplasm of overlapping sites of left bronchus and lung |
| Malignant neoplasm of unspecified part of unspecified bronchus or lung | | |
| Diagnosis | UMLS:ICD10CM:C34.9 | Malignant neoplasm of unspecified part of bronchus or lung |
| Diagnosis | UMLS:ICD10CM:C34.90 | Malignant neoplasm of unspecified part of unspecified bronchus or lung |
| Diagnosis | UMLS:ICD10CM:C34.91 | Malignant neoplasm of unspecified part of right bronchus or lung |
| Diagnosis | UMLS:ICD10CM:C34.92 | Malignant neoplasm of unspecified part of left bronchus or lung |
| Acute lower respiratory infections | | |
| Diagnosis | UMLS:ICD10CM:J20-J22 | Other acute lower respiratory infections |
| Influenza and pneumonia | | |
| Diagnosis | UMLS:ICD10CM:J09-J18 | Influenza and pneumonia |
| Suppurative and necrotic conditions of the lower respiratory tract | | |
| Diagnosis | UMLS:ICD10CM:J85-J86 | Suppurative and necrotic conditions of the lower respiratory tract |
| Pulmonary fibrosis | | |
| Diagnosis | UMLS:ICD10CM:J84.1 | Other interstitial pulmonary diseases with fibrosis |
| MAKE | | |
| Outcome definition | | |
| Diagnosis | UMLS:ICD10CM:N17 | Acute kidney failure |
| Diagnosis | UMLS:ICD10CM:N18.5 | Chronic kidney disease, stage 5 |
| Diagnosis | UMLS:ICD10CM:N18.6 | End stage renal disease |
| Demographics | Deceased | Deceased |
| Procedure | UMLS:CPT:1012740 | Dialysis Services and Procedures |
| Procedure | UMLS:CPT:90945 | Dialysis procedure other than hemodialysis (eg, peritoneal dialysis, hemofiltration, or other continuous renal replacement therapies), with single evaluation by a physician or other qualified health care professional |
| Diagnosis | UMLS:ICD10CM:Z99.2 | Dependence on renal dialysis |
| MACE | | |
| Diagnosis | UMLS:ICD10CM:I21 | Acute myocardial infarction |
| Diagnosis | UMLS:ICD10CM:I63 | Cerebral infarction |
| Demographics | Deceased | Deceased |
| Bone fracture | | |
| Diagnosis | UMLS:ICD10CM:S12 | Fracture of cervical vertebra and other parts of neck |
| Diagnosis | UMLS:ICD10CM:S22 | Fracture of rib(s), sternum and thoracic spine |
| Diagnosis | UMLS:ICD10CM:S32 | Fracture of lumbar spine and pelvis |
| Diagnosis | UMLS:ICD10CM:S42 | Fracture of shoulder and upper arm |
| Diagnosis | UMLS:ICD10CM:S52 | Fracture of forearm |
| Diagnosis | UMLS:ICD10CM:S72 | Fracture of femur |
| Diagnosis | UMLS:ICD10CM:S82 | Fracture of lower leg, including ankle |
| Diagnosis | UMLS:ICD10CM:S92 | Fracture of foot and toe, except ankle |
| Scleroderma | | |
| Diagnosis | UMLS:ICD10CM:M34 | Systemic sclerosis [scleroderma] |

## **Codes for the baseline characteristics**

Propensity score matching was performed on all listed characteristics. Characteristics of the cohorts before and after matching are summarized in the table below.

| **Characteristics after propensity score matching** | | | | |
| --- | --- | --- | --- | --- |
| **Demographics** | | | | |
| AI | Age at Index | | | |
| Male | Male | | | |
| 2106-3 | White | | | |
| 2054-5 | African American | | | |
| 2028-9 | Asian | | | |
| 2131-1 | Other | | | |
| UNK | Unknown | | | |
| 2186-5 | Not Hispanic or Latino | | | |
| 2135-2 | Hispanic or Latino | | | |
| UN | Unknown Ethnicity | | | |
| **Lifestyles** | | | | |
| International Classification of  Diseases, Tenth Revision, | Name | | | |
| Z55-Z65 | Persons with potential health hazards related to socioeconomic and psychosocial circumstances | | | |
| Z59 | Problems related to housing and economic circumstances | | | |
| Z56 | Problems related to employment and unemployment | | | |
| Z72.0 | Tobacco use | | | |
| F17 | Nicotine dependence | | | |
| F10 | Alcohol related disorders | | | |
| Z72 | Problems related to lifestyle | | | |
| **History of cancer** | | | | |
| International Classification of  Diseases, Tenth Revision, | Name | | | |
| Z80 | Family history of primary malignant neoplasm | | | |
| Z15.0 | Genetic susceptibility to malignant neoplasm | | | |
| Z85 | Personal history of malignant neoplasm | | | |
| **Diagnosis** | | | | |
| International Classification of  Diseases, Tenth Revision, | Name | | | |
| I10-I1A | Hypertensive diseases | | | |
| I50 | Heart failure | | | |
| I20-I25 | Ischemic heart diseases | | | |
| E78 | Disorders of lipoprotein metabolism and other lipidemias | | | |
| E66 | Overweight and obesity | | | |
| I60-I69 | Cerebrovascular diseases | | | |
| I70-I79 | Diseases of arteries, arterioles and capillaries | | | |
| J45 | Asthma | | | |
| N18 | Chronic kidney disease (CKD) | | | |
| J44 | Other chronic obstructive pulmonary disease | | | |
| J96 | Respiratory failure, not elsewhere classified | | | |
| J84 | Other interstitial pulmonary diseases | | | |
| J84.10 | Pulmonary fibrosis, unspecified | | | |
| Z94 | Transplanted organ and tissue status | | | |
| **Medication** | | | | |
| Anatomical Therapeutic  Chemical | Name | | | |
| A10A | INSULINS AND ANALOGUES | | | |
| A10BA | Biguanides | | | |
| A10BK | Sodium-glucose co-transporter 2 (SGLT2) inhibitors | | | |
| A10BB | Sulfonylureas | | | |
| A10BG | Thiazolidinediones | | | |
| A10BX | Other blood glucose lowering drugs, excl. insulins | | | |
| A10BF | Alpha glucosidase inhibitors | | | |
| C10A | LIPID MODIFYING AGENTS, PLAIN | | | |
| C09A | ACE INHIBITORS, PLAIN | | | |
| C09C | ANGIOTENSIN II RECEPTOR BLOCKERS (ARBs), PLAIN | | | |
| C07 | BETA BLOCKING AGENTS | | | |
| C08 | CALCIUM CHANNEL BLOCKERS | | | |
| C03 | DIURETICS | | | |
| N05C | HYPNOTICS AND SEDATIVES | | | |
| CN104 | NON-STEROIDAL ANTI-INFLAMMATORY ANALGESICS | | | |
| **Laboratory** | | | | |
| TNX Curated | Name | Unit | Missing rate | |
|  | | | GLP-1 RA users | DPP4i users |
| 9029 | Sodium | mmol/L | 44.70% | 44.90% |
| 9014 | Hemoglobin | g/dL | 54.70% | 54.60% |
| 9044 | Alanine aminotransferase | U/L | 50.60% | 50.80% |
| 9047 | Aspartate aminotransferase | U/L | 51.00% | 51.20% |
| 9002 | Cholesterol in LDL | mg/dL | 56.60% | 56.70% |
| 9004 | Triglyceride | mg/dL | 55.60% | 55.80% |
| 9037 | Hemoglobin A1c | % | 49.40% | 50.60% |
| 9083 | BMI | kg/m2 | 58.20% | 58.20% |
| 9045 | Albumin | g/dL | 53.40% | 53.40% |
| LG34557-5 | Albumin/Creatinine in Urine | ug/mg | 95.60% | 95.70% |
| 62238-1 | Glomerular filtration rate/1.73 (CKD-EPI) | mL/min/  {1.73_m2} | 56.60% | 56.70% |
| 9046 | Alkaline phosphatase | U/L | 52.30% | 52.50% |

**eMethod 2** The detail of propensity score matching in TriNetX platform

**(https://support.trinetx.com/hc/en-us/articles/360011978033)**

To conduct PSM using the TNX Research platform, you must first identify two cohorts of interest, index events, outcomes of interest, and attributes of patients which may act as confounders to the outcomes of interest. For the purpose of this article, these attributes will be called “covariates.”

Within Balance, when you run a propensity score matching analysis, the system conducts a propensity score matching to balance the cohorts:

1. For each patient in each cohort, the system computes values for each covariate.
2. These data form a matrix of covariate values for each
3. The system performs a logistic regression on the pooled matrices, to “predict” which cohort each patient originates from. The value of this model for a patient is that patient’s predicted probability of being in the second cohort or “propensity score.”
4. For each patient in the smaller cohort, the system chooses as match from the larger cohort (if any patients in the larger cohort are close enough). The pairs then form a subset of each cohort.

Within Outcomes, when you run compare cohorts after matching, the system conducts a propensity score matching to compare outcomes between the balance the cohorts:

1. For each patient in each cohort, the system computes both the outcome(s) of interest and values for each covariate.
2. These data form a matrix of covariate values for each
3. The system performs a logistic regression on the pooled matrices, to “predict” which cohort each patient originates from. The value of this model for a patient is that patient’s predicted probability of being in the second cohort or “propensity score.”
4. For each patient in the smaller cohort, the system chooses as match from the larger cohort (if any patients in the larger cohort are close enough). The pairs then form a subset of each cohort.
5. The system compares outcomes on these after matching subsets, rather than the original cohorts.

**Values in the Covariate Matrix**

In the covariate matrix, each row represents one patient, and each column represents one covariate. Each cell contains exactly one non-null numerical value.

 All covariates are one of the following forms:

- **Binary:** yes/no;
- **Categorical:** real values placed in categories based on their value; or
- **Continuous:** real values represented directly.

When a covariate is binary (for example, a patient having history of asthma), the cell for that patient and covariate is set to 0 for “not present” or 1 for “is present.”

When a covariate is categorical, the categories are specified as ranges that the continuous variable can take (for example, having a value for a Sodium lab between 130 and 140). Each category becomes a distinct covariate which is either 0 for “not present” or 1 for “is present.” It is possible for all columns to be 0 (for example if there are no values in the time window, or if all values are outside all the categories). It is also possible for multiple values to be 1 (for example if there are multiple values in the time window, or if the categories overlap).

When a covariate is continuous (for example, current age, or age at time of the index event), then the value for that patient (if any) is placed in the relevant cell.

**Details on Scoring and Logistic Regression**

When you run propensity score matching, the system generates a propensity score for each patient in each cohort. The propensity score ranges between 0 and 1 and indicates the predicted probability a patient is in cohort B given the patient’s covariates.

To uses logistic regression to generate the propensity scores, through an implementation of the well-tested, standard software package [scikit-learn](https://scikit-learn.org/). The code used in production is as follows (some logging, etc. removed for clarity):

import numpy as np

from sklearn.linear_model import LogisticRegression

from sklearn.preprocessing import Imputer

from impute import fill_nans

def fill_nans(data, axis=0):

  imp = Imputer(missing_values='NaN', strategy=method, axis=axis)

    return imp.fit_transform(data)

def propensity_scores(matrix_a, matrix_b):

matrix_a = fill_nans(matrix_a, method='mean')

matrix_b = fill_nans(matrix_b, method='mean')

full_matrix = np.concatenate((matrix_a, matrix_b), axis=0)

# cohort A is "zero" and cohort B is "one" from the regression’s perspective

target_a = np.zeros(matrix_a.shape[0], dtype=float)

target_b = np.ones(matrix_b.shape[0], dtype=float)

target = np.concatenate([target_a, target_b])

lr = LogisticRegression(C=1000)

lr.fit(full_matrix, target)

def get_scores(X):

# .predict_proba returns [prob_class_zero, prob_class_one] for each row,

# while the score we want is the probability of being in class B

full_scores = lr.predict_proba(X)

return full_scores[:, 1]

return (get_scores(matrix_a), get_scores(matrix_b))

Here the “score” for each patient is the regression model applied to that patient’s row. Informally, this is the probability that the patient belongs to cohort B, based on the cohorts it has seen.

Here “matrix_a” is the covariate matrix for the cohort you selected as Cohort A. Likewise, “matrix_b” is the covariate matrix for the cohort you selected as Cohort B.  Data are pooled across all HCOs, so all patients in the analysis are a row in one of these matrices. Note that the code instructs the system to replace all missing (NaN) values in each matrix with the mean from that column; however, at time of writing all covariates are either binary, categorical (which expands to a set of binary columns), or continuous but guaranteed to exist, so this imputation is vacuous.

The call “LogisticRegression(C=1000)” means we predict the probability of being in cohort B using logistic regression. The value of C means we weight the mean residual by a factor of 1000 as compared to the norm of the coefficients. This means we are using a very small amount of L2 (ridge) regression, which is needed to make the objective function convex (so the regression converges), but with such a small value, it has very little effect on the model. Due to the very small amount of regularization and the scale of our covariates being constrained, it is not necessary to perform mean normalization as a preprocessing step.

**Matching Details**

Once the system has generated a propensity score for each patient, the system performs matching to identify the matched subsets. We use “greedy nearest neighbor matching” with a caliper of 0.1 pooled standard deviations. Sample code is here:

import numpy as np

def _pooled_sd(floats_a, floats_b):

var_a = np.var(floats_a)

var_b = np.var(floats_b)

return sqrt((var_a + var_b) / 2.0)

def _naive_nearest_neighbor_match(scores_a, scores_b, caliper):

# Perform nearest neighbor matching. Assume that len(scores_a) <=len(scores_b)

num_a, num_b = len(scores_a), len(scores_b)

max_diff = _pooled_sd(scores_a, scores_b) * caliper

chosen_a = np.zeros(num_a, dtype=bool) # True if the patient is matched, start false

chosen_b = np.zeros(num_b, dtype=bool)

for a_ind in range(0, num_a):

best_diff = max_diff

best_b_ind = None

found_any = False

for b_ind in range(0, num_b):

if chosen_b[b_ind]:

continue

diff = abs(scores_a[a_ind] - scores_b[b_ind])

if diff < best_diff or (diff == best_diff and not found_any):

best_diff = diff

best_b_ind = b_ind

found_any = True

if best_b_ind is not None:

chosen_a[a_ind] = True

chosen_b[best_b_ind] = True

return (chosen_a, chosen_b)

*Note:* the code used in production is highly optimized and significantly longer. The above code is taken directly from our test fixtures and is used to ensure that the optimized code produces identical results to the above simplified code.

We use a “caliper” of 0.1 pooled standard deviations of the propensity scores in aggregate, which means that patients with very different propensity scores are not matched. The system matches patients using the following algorithm:

1. For each patient in cohort A (assumed to be the smaller of the two), and identify the patient whose score is closest to the patient in cohort A from the patients in cohort B who have not yet been matched.
2. If a match is found, mark both as “chosen” and move on.
3. At the end, return the labels of each cohort, indicating which patients were chosen and which were not. The patients who were chosen form the “matched” cohorts.

**Pooling the Cohorts, and Compensating for Unbalanced Cohorts across HCOs**

TriNetX pulls data from a federated data network made up of many healthcare organizations across the world. Each site computes a covariate matrix for the patients they contribute to the analysis and send it to a central processing point to be pooled and analyzed as a single matrix.

The order of the rows in the matrix should not impact the propensity scores generated for each patient; logistic regression is highly stable with respect to permuting the rows.  In contrast, nearest neighbor matching, can be influenced by the order of rows in the matrix. For example, if two identical patients in cohort B match equally well to a patient in cohort A, the first will be chosen and the second will not.  Therefore, if the order of the rows carries some information (perhaps as an artifact of the pooling), the order can introduce bias.

To eliminate this bias, we randomize the order of the records in the covariate matrix.  We sort the provider results by the unique ID of the HCO (so the information of “which HCO responded first” is deleted), concatenate the matrices into one large matrix, then shuffle them using np.random.shuffle(full_matrix_a) and np.random.shuffle(full_matrix_b). To assure determinism, a call to np.random.seed(FIXED_SEED) precedes all calls to shuffle, so that successive runs do not change unless the underlying data changes.

**eFigure1.** Kaplan–Meier plots of lung cancer


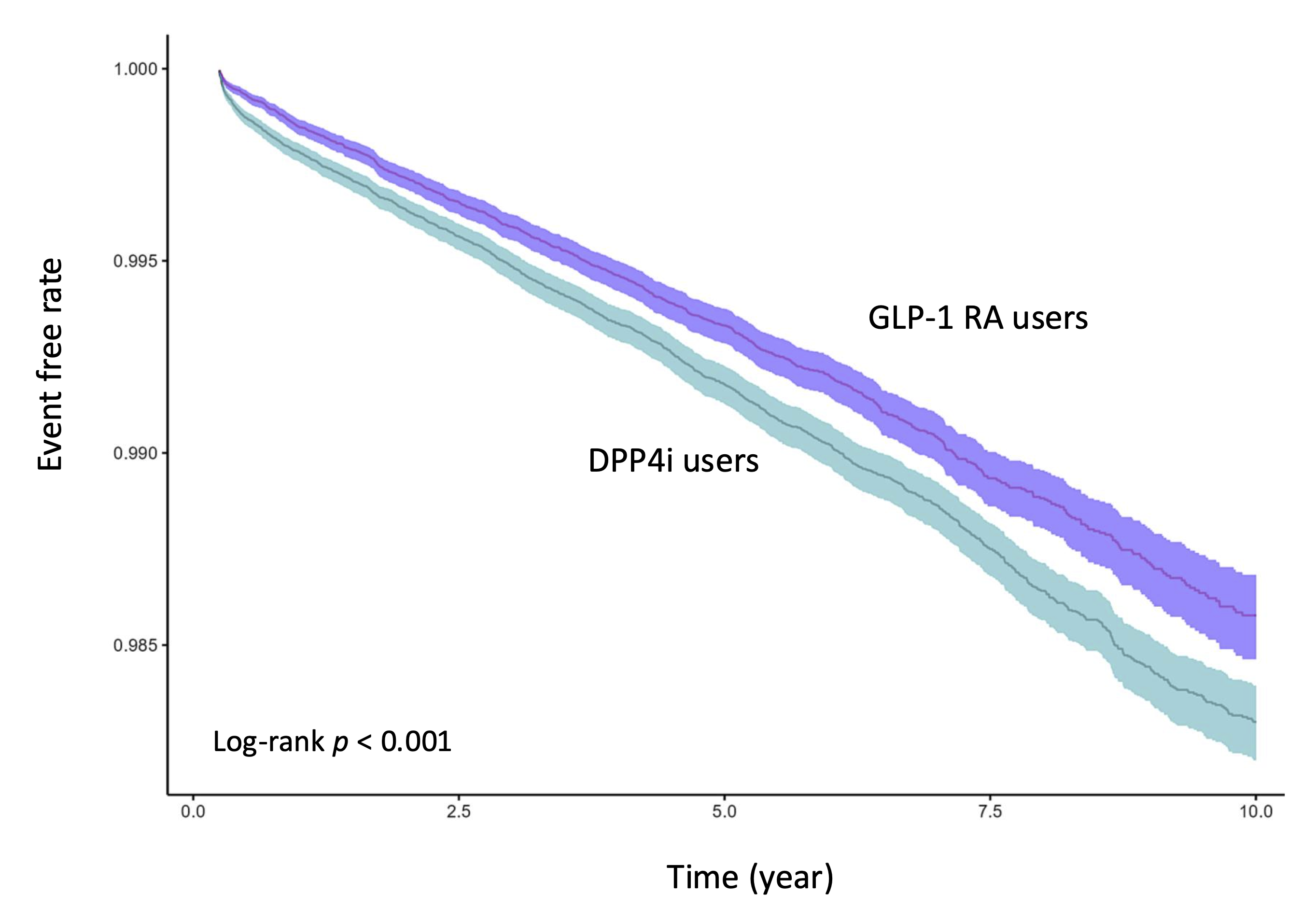


The cumulative event-free plots of lung cancer were compared between GLP-1 RA and DPP4i users. Shaded areas indicate 95% confidence intervals.

**Abbreviations:** GLP-1 RA, glucagon-like peptide-1 receptor agonist; DPP4i, dipeptidyl peptidase-4 inhibitor.


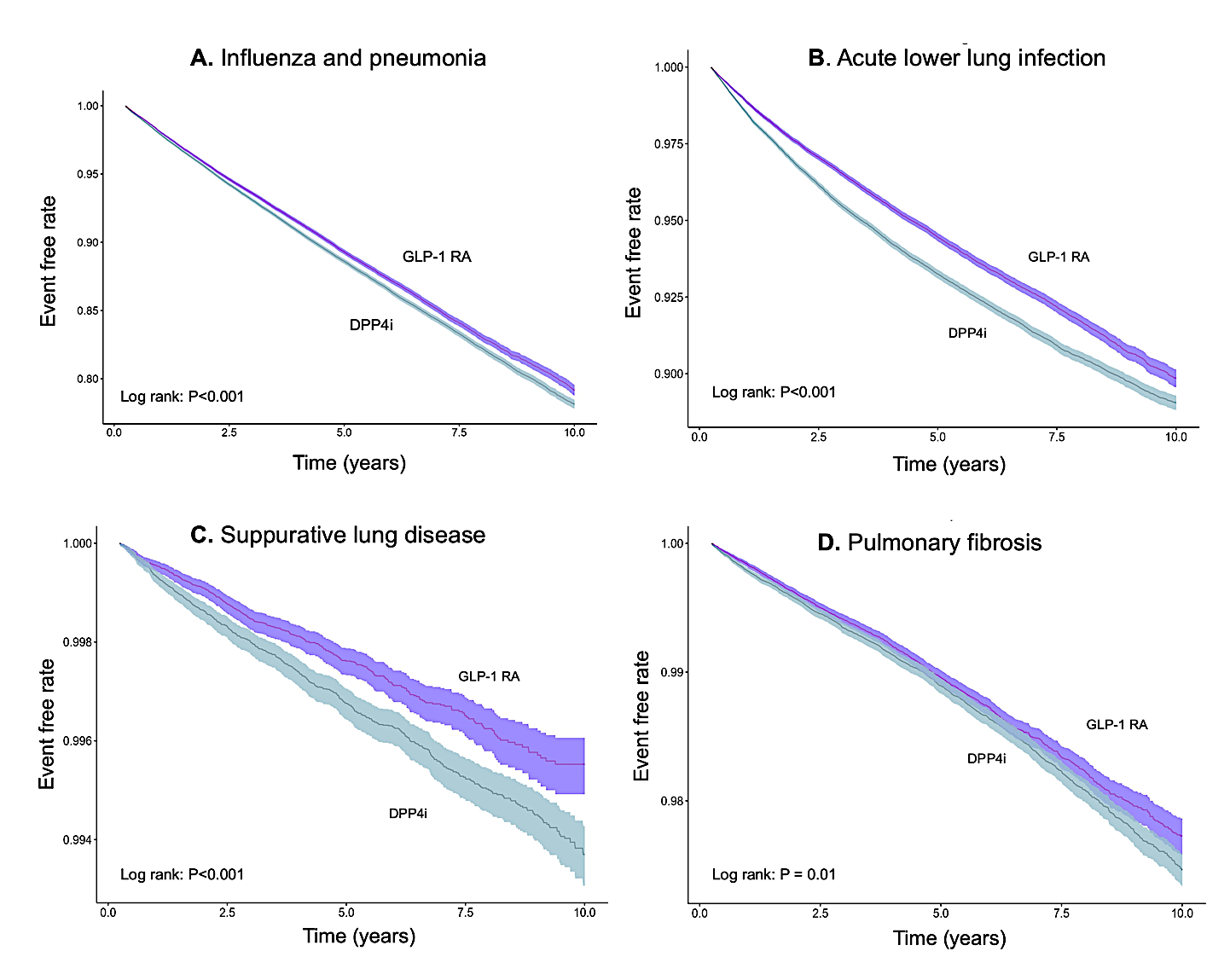
**eFigure 2.** Kaplan–Meier plots of pulmonary infection and fibrosis

The cumulative event-free plots of (A) influenza and pneumonia, (B) acute lower lung infection, (C) suppurative lung disease, and (D) pulmonary fibrosis were compared between GLP-1 RA and DPP4i users. Shaded areas indicate 95% confidence intervals.

**Abbreviations:** GLP-1 RA, glucagon-like peptide-1 receptor agonist; DPP4i, dipeptidyl peptidase-4 inhibitor.

**eFigure 3.** Subgroup analysis for influenza and pneumonia

**
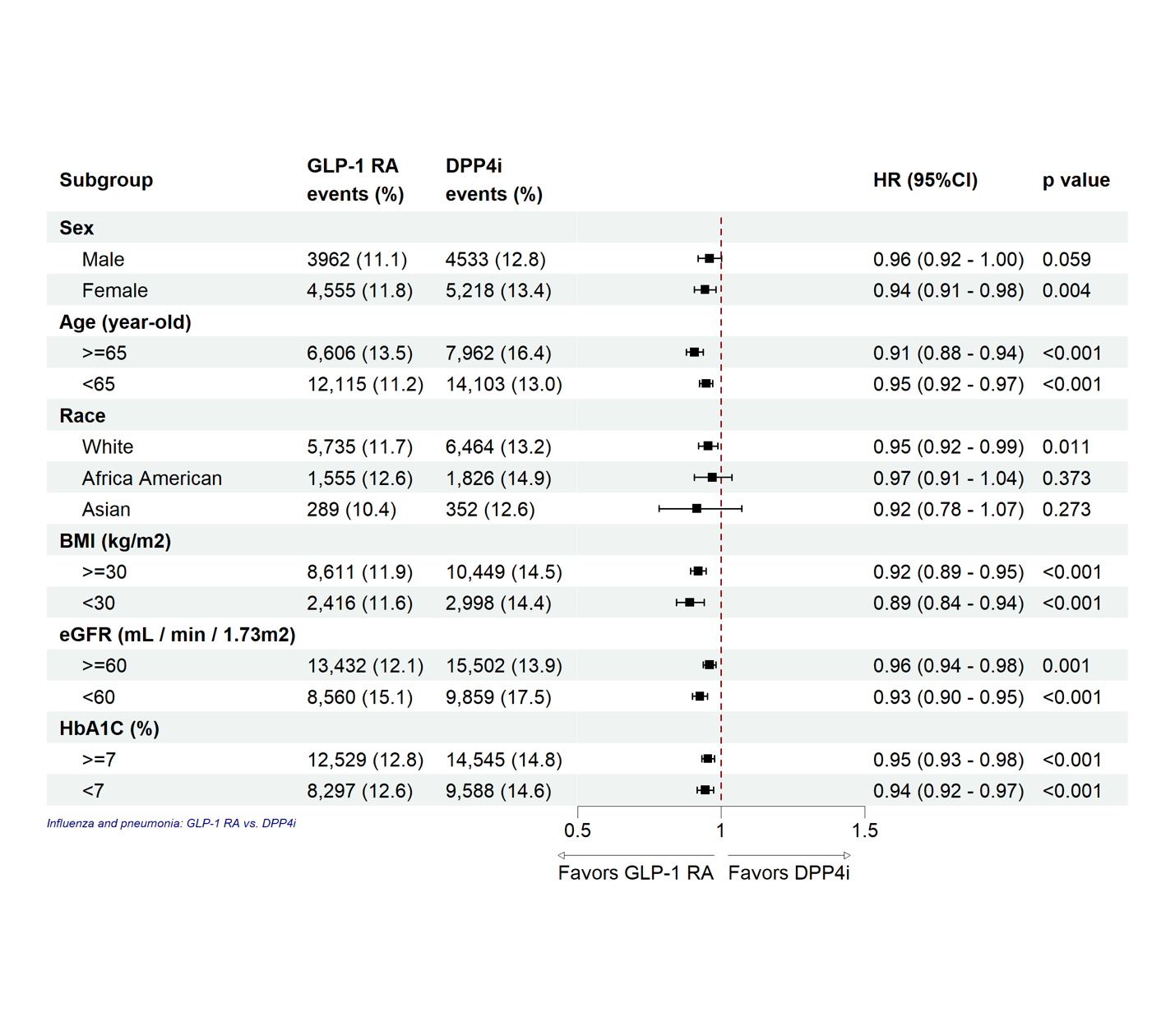
**

The vertical line indicates an HR of 1.00. A lower limit of the 95% CI greater than 1.00 indicates a significantly higher risk.

**Abbreviations:** GLP1-RA, glucagon-like peptide-1 receptor agonist; DPP4i, dipeptidyl peptidase-4 inhibitor; HR, hazard ratio; CI, confidence interval; BMI, body mass index; eGFR, estimated glomerular filtration rate; HbA1c, glycated hemoglobin.

**eFigure 4**. Subgroup analysis for acute lower lung infection

**
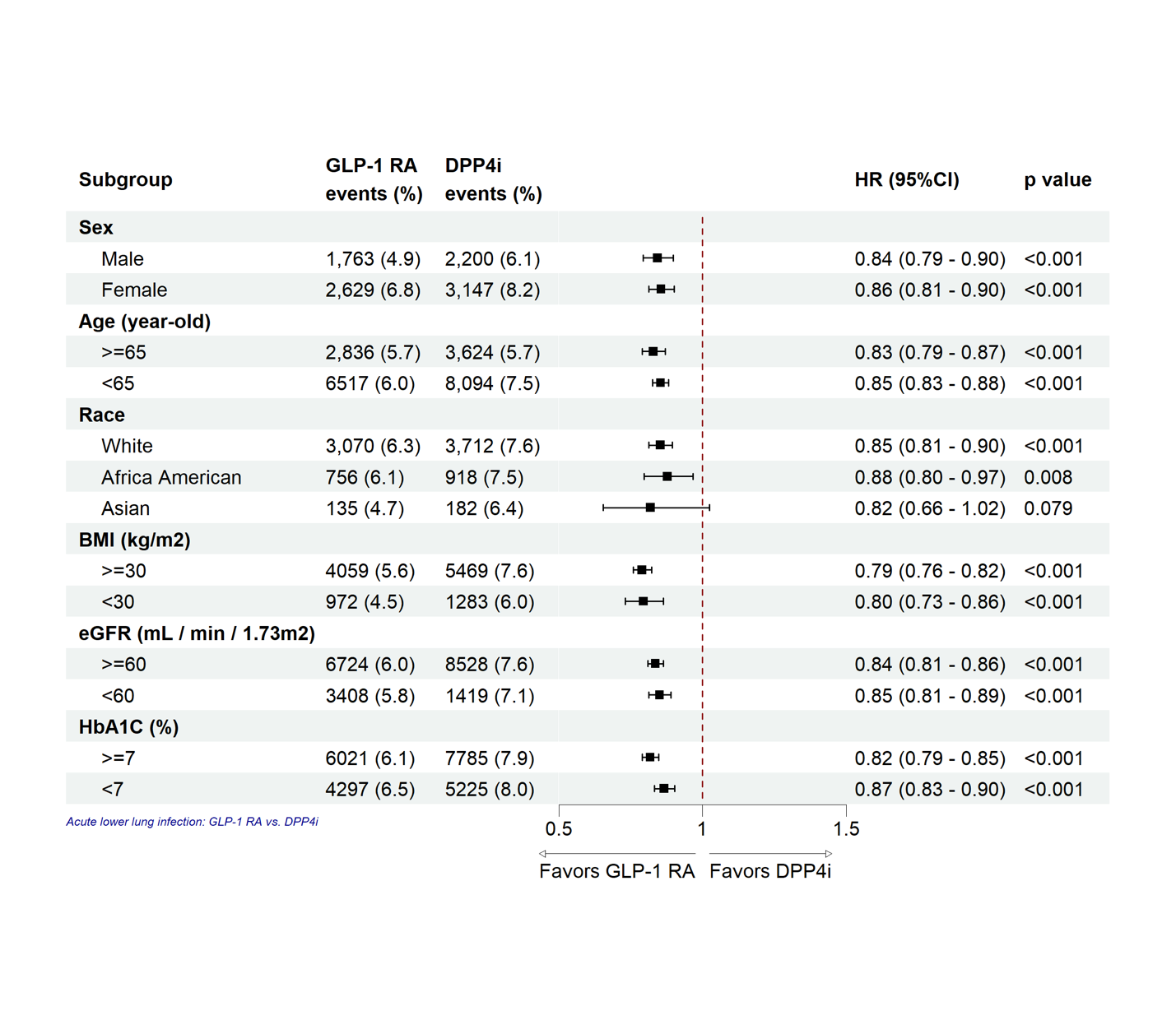
**

The vertical line indicates an HR of 1.00. A lower limit of the 95% CI greater than 1.00 indicates a significantly higher risk.

**Abbreviations:** GLP1-RA, glucagon-like peptide-1 receptor agonist; DPP4i, dipeptidyl peptidase-4 inhibitor; HR, hazard ratio; CI, confidence interval; BMI, body mass index; eGFR, estimated glomerular filtration rate; HbA1c, glycated hemoglobin.

**eFigure 5**. Subgroup analysis for suppurative lung disease

**
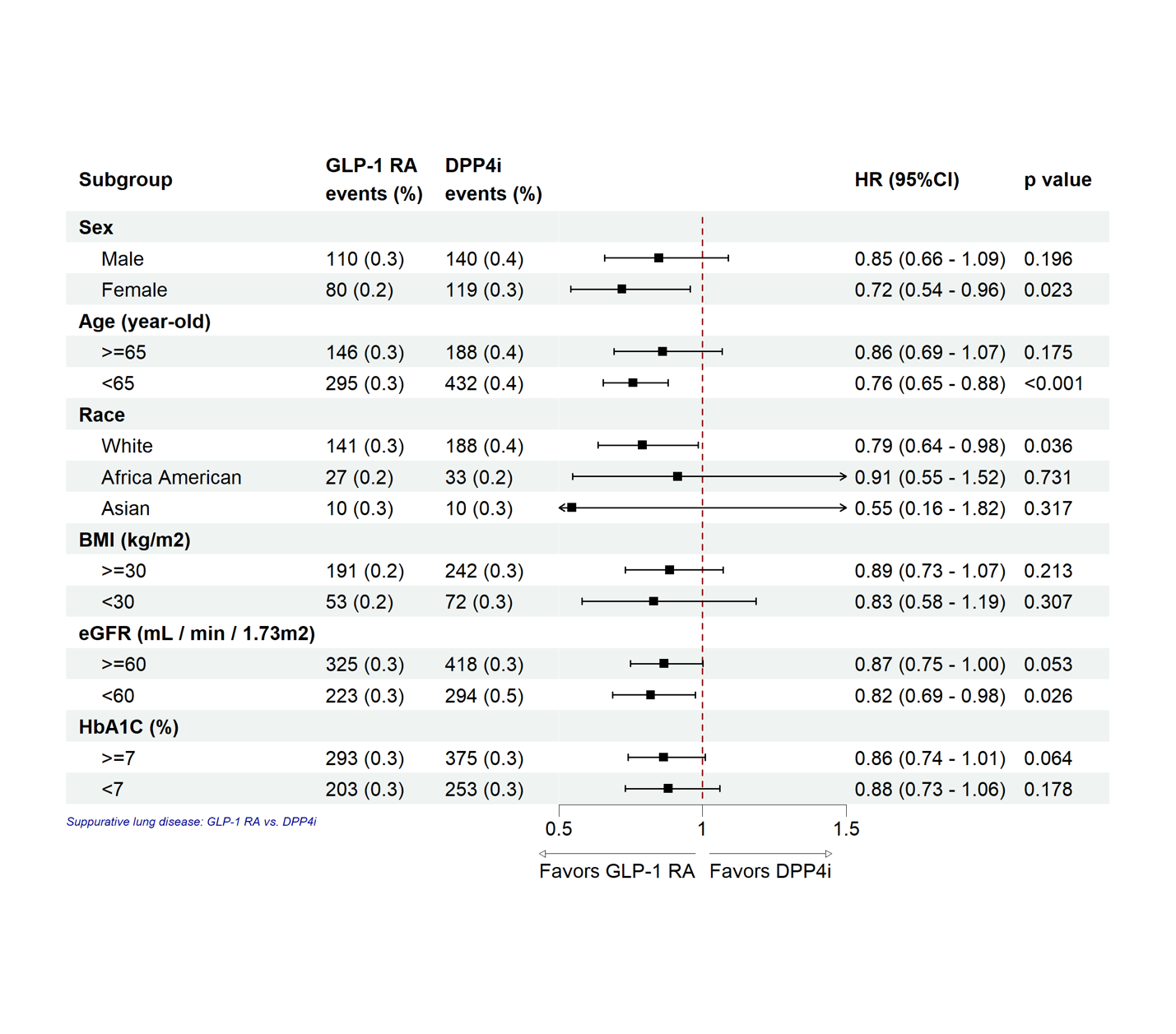
**

The vertical line indicates an HR of 1.00. A lower limit of the 95% CI greater than 1.00 indicates a significantly higher risk.

**Abbreviations:** GLP1-RA, glucagon-like peptide-1 receptor agonist; DPP4i, dipeptidyl peptidase-4 inhibitor; HR, hazard ratio; CI, confidence interval; BMI, body mass index; eGFR, estimated glomerular filtration rate; HbA1c, glycated hemoglobin.

**
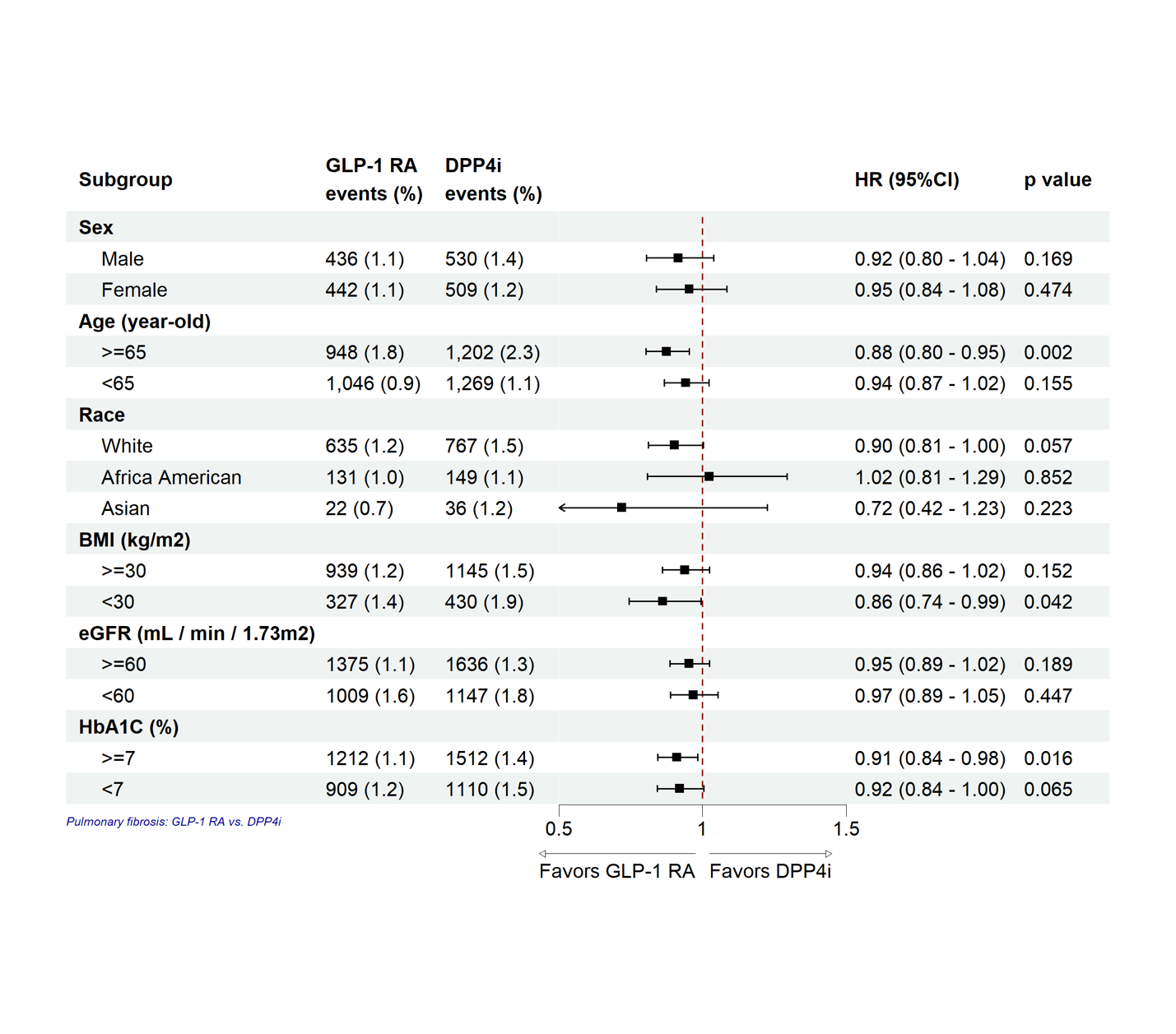
eFigure 6**. Subgroup analysis for pulmonary fibrosis

The vertical line indicates an HR of 1.00. A lower limit of the 95% CI greater than 1.00 indicates a significantly higher risk.

**Abbreviations:** GLP1-RA, glucagon-like peptide-1 receptor agonist; DPP4i, dipeptidyl peptidase-4 inhibitor; HR, hazard ratio; CI, confidence interval; BMI, body mass index; eGFR, estimated glomerular filtration rate; HbA1c, glycated hemoglobin.

| **eTable 1.** The positive and negative outcome control | | | | |
| --- | --- | --- | --- | --- |
| **Clinical Outcomes** | GLP-1 RA user | DPP4i user | GLP-1 RA user vs. DPP4i user | |
|  | Events/number at risk^#^ (%) | | HR (95%CI) | *P* Value |
| **Positive control** |  |  |  |  |
| Major adverse cardiac events | 21,151/45342 (14.5) | 25,049/144,899(17.3) | 0.94 (0.93–0.96) | <0.001 |
| Major adverse kidney events | 25,986/143,973 (18.0) | 30,022/142,231(21.1) | 0.96 (0.94–0.97) | <0.001 |
| **Negative control** |  |  |  |  |
| Bone fracture | 12,205/149,530 (8.2) | 13,524/149,760 (9.0) | 1.01 (0.99–1.04) | 0.274 |
| Scleroderma | 121/158,099 (0.1) | 122/158,102 (0.1) | 1.08 (0.84–1.39) | 0.546 |
| ^#^ The number at risk refers to patients who have not experienced the outcome before follow-up begins.  Major adverse cardiovascular events include cerebral infarction, myocardial infarction, and death; major adverse kidney events include acute and advanced kidney failure, initiation of dialysis, and death.  **Abbreviation :** GLP1-RA, glucagon-like peptide-1 receptor agonists**;** DPP4i, dipeptidyl peptidase 4 inhibitor ; HR, hazard ratio; CI, confidence interval; | | | | |

| **eTable 2 .** Sensitivity test by extending the index date by 6 months | | | | |
| --- | --- | --- | --- | --- |
| Clinical Outcomes | After Matching | | | |
|  | GLP-1 RA users | DDP-4i users | GLP-1 RA vs. DDP-4i users | |
|  | Events / number at risk^#^ (%) | | HR (95%CI) | *p* value |
| **Primary outcomes** |  |  |  |  |
| Lung cancer | 614/ 87,388 (0.7) | 777/ 87,388 (0.9) | 0.86 (0.77–0.95) | 0.005 |
| **Secondary outcomes** |  |  |  |  |
| Influenza and pneumonia | 8,539/80,490 | 9,666/80,596 | 0.96 (0.93–0.99) | 0.004 |
| Other acute lower lung infection | 4,245/81,600 (5.2) | 4,576/81,511 (5.6) | 0.98 (0.94–1.02) | 0.383^†^ |
| Suppurative lung disease | 192/87,149 (0.2) | 298/87,116 (0.3) | 0.70 (0.59–0.84) | <0.001 |
| Pulmonary fibrosis | 929/86,845 (1.1) | 1,109/86844 (1.3) | 0.92 (0.84–0.99) | 0.049 |
| ^#^ The number at risk refers to patients who have not experienced the outcome before follow-up begins.  ^†^indicates a violation of the proportional hazard’s assumption in Cox regression.  **Abbreviation:** GLP-1 RA, glucagon-like peptide-1 receptor agonist; DDP-4i, dipeptidyl peptidase-4 inhibitor; HR, hazard ratio; CI, confidence interval | | | | |

| **eTable 3 .** Sensitivity test by extending the index date by 12 months | | | | |
| --- | --- | --- | --- | --- |
| Clinical Outcomes | After Matching | | | |
|  | GLP-1 RA users | DDP-4i users | GLP-1 RA vs. DDP-4i users | |
|  | Events / number at risk^#^ (%) | | HR (95%CI) | *p* value |
| **Primary outcomes** |  |  |  |  |
| Lung cancer | 1,191/183,092 (0.65) | 1,553/182,974 (0.85) | 0.86 (0.80–0.92) | <0.001 |
| **Secondary outcomes** |  |  |  |  |
| Influenza and pneumonia | 18,851/166,479 (11.32) | 22,186/165,990 (13.37) | 0.95 (0.94–0.97) | <0.001 |
| Other acute lower lung infection | 9,115/169,207 (5.39) | 11,355/167,901 (6.76) | 0.86 (0.84–0.89) | <0.001^†^ |
| Suppurative lung disease | 440/182,893 (0.24) | 602/182,806 (0.33) | 0.83 (0.73–0.94) | 0.002 |
| Pulmonary fibrosis | 2,002/181,986 (1.10) | 2,382/181,939 (1.31) | 0.96 (0.91–1.02) | 0.215 |
| ^#^ The number at risk refers to patients who have not experienced the outcome before follow-up begins.  ^†^indicates a violation of the proportional hazard’s assumption in Cox regression.  **Abbreviation:** GLP-1 RA, glucagon-like peptide-1 receptor agonist; DDP-4i, dipeptidyl peptidase-4 inhibitor; HR, hazard ratio; CI, confidence interval | | | | |

| **eTable 4.** Propensity score models for confounding adjusting | | | | | | |
| --- | --- | --- | --- | --- | --- | --- |
| Clinical Outcomes | GLP-1 RA vs. DPP4i with a 10-year follow-up | | | | | |
|  | Model 1 | | Model 2 | | Model 3 | |
|  | HR (95%CI) | *p* value | HR (95%CI) | *p* value | HR (95%CI) | *p* value |
| **Primary outcomes** |  |  |  |  |  |  |
| Lung cancer | 0.76 (0.71, 0.82) | <0.001 | 0.82 (0.77, 0.89) | <0.001† | 0.82 (0.76, 0.88) | <0.001 |
| Trachea | 0.71 (0.25, 1.96) | 0.501 | 0.71 (0.26, 1.96) | 0.501 | 0.67 (0.24, 1.85) | 0.434 |
| Bronchus and Lung | 0.82 (0.77, 0.89) | <0.001 | 0.88 (0.82, 0.95) | 0.001 | 0.89 (0.82, 0.96) | 0.003 |
| **Secondary outcomes** |  |  |  |  |  |  |
| Influenza and pneumonia | 0.92 (0.90–0.94) | <0.001 | 0.93 (0.91–0.95) | <0.001 | 0.90 (0.89–0.92) | <0.001 |
| Other acute lower lung infection | 0.79 (0.77–0.81) | <0.001^†^ | 0.79 (0.76–0.81) | <0.001^†^ | 0.749 (0.77–0.81) | <0.001^†^ |
| Suppurative lung disease | 0.75 (0.67–0.84) | <0.001 | 0.77 (0.69–0.87) | <0.001 | 0.77 (0.68–0.87) | <0.001 |
| Pulmonary fibrosis | 0.87 (0.82–0.92) | <0.001 | 0.89 (0.83–0.94) | <0.001 | 0.88 (0.82–0.93) | <0.001 |
| Model 1 adjusts for age, sex, race, ethnicity, lifestyles, body mass index in the propensity score matching. Model 2 includes Model 1 plus baseline comorbidities, while Model 3 adds baseline medication use to Model 2 in the propensity score matching.  ^†^Indicates a p-value below 0.05 for the proportional hazard assumption.  Abbreviation: GLP-1 RA, glucagon-like peptide-1 receptor agonist; DDP-4i, dipeptidyl peptidase-4 inhibitor; HR, hazard ratio; CI, confidence interval. | | | | | | |

| **eTable 5.** Segmenting the follow-up into four periods (3 months to 3-, 5-, and 7-years post-index date) | | | | | | |
| --- | --- | --- | --- | --- | --- | --- |
| Clinical Outcomes | After matching; GLP-1 RA vs. DDP-4i users | | | | | |
|  | 3 months to 3 years | | 3 months to 5 years | | 3 months to 7 years | |
|  | HR (95% CI) | *p* value | HR (95% CI) | *p* value | HR (95% CI) | *p* value |
| **Primary outcomes** |  |  |  |  |  |  |
| Lung cancer | 0.81 (0.73– 0.90) | <0.001 | 0.84 (0.77– 0.91) | <0.001 | 0.83 (0.76– 0.89) | <0.001^†^ |
| Trachea | 0.50 (0.15–1.65) | 0.243 | 0.61 (0.22–1.66) | 0.325 | 0.79 (0.27–2.27) | 0.658 |
| Bronchus and Lung | 0.88 (0.79–0.99) | 0.030 | 0.89 (0.81–0.97) | 0.008 | 0.89 (0.82–0.97) | 0.008 |
| **Secondary outcomes** |  |  |  |  |  |  |
| Influenza and pneumonia | 0.93 (0.90–0.96) | <0.001 | 0.93 (0.91–0.95) | <0.001 | 0.92 (0.90–0.94) | <0.001^†^ |
| Other acute lower lung infection | 0.76 (0.74–0.79) | <0.001 | 0.80 (0.77–0.82) | <0.001^†^ | 0.79 (0.77–0.81) | <0.001^†^ |
| Suppurative lung disease | 0.78 (0.66–0.92) | 0.004 | 0.77 (0.67–0.89) | <0.001 | 0.80 (0.70–0.92) | 0.001 |
| Pulmonary fibrosis | 0.87 (0.80–0.96) | 0.003 | 0.91 (0.85–0.98) | 0.012 | 0.88 (0.82–0.94) | <0.001 |
| ^†^Indicates a p-value below 0.05 for the proportional hazard assumption.  **Abbreviation:**  GLP-1 RA, glucagon-like peptide-1 receptor agonist; DDP-4i, dipeptidyl peptidase-4 inhibitor; HR, hazard ratio; CI, confidence interval. | | | | | | |
